# Polyester Adhesives via One-Pot, One-Step Copolymerization of Cyclic Anhydride, Epoxide, and Lactide

**DOI:** 10.3390/polym16192767

**Published:** 2024-09-30

**Authors:** Ryota Suzuki, Toshiki Miwa, Ryosuke Nunokawa, Ayaka Sumi, Masaru Ando, Katsuaki Takahashi, Akira Takagi, Takuya Yamamoto, Kenji Tajima, Feng Li, Takuya Isono, Toshifumi Satoh

**Affiliations:** 1 Graduate School of Chemical Sciences and Engineering, Hokkaido University, Sapporo 060-8628, Japan; suzuki.ryota.k4@elms.hokudai.ac.jp (R.S.); miwa.toshiki.y9@elms.hokudai.ac.jp (T.M.); nunosuke2001@eis.hokudai.ac.jp (R.N.); 2 Division of Applied Chemistry, Faculty of Engineering, Hokkaido University, Sapporo 060-8628, Japan; a.sumi@eng.hokudai.ac.jp (A.S.); yamamoto.t@eng.hokudai.ac.jp (T.Y.); ktajima@eng.hokudai.ac.jp (K.T.); feng.li@eng.hokudai.ac.jp (F.L.); 3 Toagosei Co., Ltd., Nagoya 455-0026, Japan; masaru_andou@mail.toagosei.co.jp (M.A.); katsuaki_takahashi@mail.toagosei.co.jp (K.T.); akira_takagi@mail.toagosei.co.jp (A.T.); 4 List Sustainable Digital Transformation Catalyst Collaboration Research Platform (ICReDD List-PF), Institute for Chemical Reaction Design and Discovery, Hokkaido University, Sapporo 001-0021, Japan; 5 Department of Chemical & Materials Engineering, National Central University, Taoyuan 320317, Taiwan

**Keywords:** polyester, self-switchable polymerization, hot-melt adhesives

## Abstract

Polyesters (PEs) are sustainable alternatives for conventional polymers owing to their potential degradability, recyclability, and the wide availability of bio-based monomers for their synthesis. Herein, we used a one-pot, one-step self-switchable polymerization linking the ring-opening alternating copolymerization (ROAC) of epoxides/cyclic anhydrides with the ring-opening polymerization (ROP) of L-lactide (LLA) to synthesize PE-based hot-melt adhesives with a high bio-based content. In the cesium pivalate-catalyzed self-switchable polymerization of glutaric anhydride (GA), butylene oxide (BO), and LLA using a diol initiator, the ROAC of GA and BO proceeded whereas the ROP of LLA simultaneously proceeded very slowly, resulting in a copolyester consisting of poly(GA-*alt*-BO) and poly(L-lactide) (PLLA) segments with tapered regions, that is, PLLA-*tapered block*-poly(GA-*alt*-BO)-*tapered block*-PLLA (PLLA-*tb*-poly(GA-*alt*-BO)-*tb*-PLLA). Additionally, a series of tapered-block or real-block copolyesters consisting of poly(anhydride-*alt*-epoxide) (A segment) and PLLA (B segment) with AB-, BAB-, (AB)_3_-, and (AB)_4_-type architectures of different compositions and molecular weights were synthesized by varying the monomer combinations, alcohol initiators, and initial feed ratios. The lap shear tests of these copolyesters revealed an excellent relationship between the adhesive strength and polymer structural parameters. The (AB)_4_-type star-block copolyester (poly(GA-*alt*-BO)-*tb*-PLLA)_4_ exhibited the best adhesive strength (6.74 ± 0.64 MPa), comparable to that of commercial products, such as PE-based and poly(vinyl acetate)-based hot-melt adhesives.

## 1. Introduction

Polymer products such as plastics, elastomers, and fibers are essential for daily life. However, most of these polymer materials are derived from petroleum-based monomers such as styrene, ethylene, and propylene. Because lowering our dependence on petroleum resources is essential for creating a sustainable society, interest in sustainable plastics as a solution to this pervasive global problem has been increasing in recent years, with polyester (PE) emerging as one of the most sustainable polymers. Poly(lactide) (PLA) is a well-known sustainable polymer material because it is derived from renewable resources and is biodegradable. However, a significant drawback of PLA is its brittleness, which limits its industrial application as a sustainable alternative to conventional petroleum-based polymers. Therefore, the development of a new strategy for improving the physical properties of PLA is urgently needed to expand its possible application range.

Block copolymerization is an attractive approach for enhancing the properties and functionalities of polymers. For example, block copolymers consisting of PLA and poly(*ε*-caprolactone) (PCL) have been studied as thermoplastic elastomers, compatibilizers, and shape memory materials [1,2,3,4,5,6]. Additionally, combining PLA with other bio-based PEs, e.g., polymenthide and poly(*γ*-methyl-*ε*-caprolactone), has also been investigated for various applications, including pressure-sensitive adhesives and tough plastics [7,8].

To further expand the potential of PLA through block copolymerization, we used ring-opening alternating copolymerization (ROAC) to synthesize counter PE blocks. In recent years, the ROAC of cyclic anhydrides and epoxides has attracted attention as a useful method for the precise synthesis of PEs [9,10,11,12,13,14,15]. As ROAC arranges the two types of monomers in an alternating sequence, it readily produces PEs with diverse repeating structures, realizing tunable properties depending on the combination of the two monomers. Because of this advantage, ROAC has recently been applied to the design of novel PE-based materials. For example, Williams et al. reported the synthesis of BAB-type triblock copolymers composed of poly(*ε*-decalactone) (PDL; A soft segment) and poly(anhydride-*alt*-epoxide) (B hard segment), i.e., poly(anhydride-*alt*-epoxide)-*b*-PDL-*b*-poly(anhydride-*alt*-epoxide), through a one-pot, two-step monomer addition method and demonstrated their use as pressure-sensitive adhesives [16]. Similarly, the same group successfully developed thermoplastic elastomers using the ROAC approach [17,18]. Therefore, linking diverse PE syntheses via ROAC with the development of PLA-based block copolyesters can lead to the creation of novel functional sustainable polymer materials. 

Self-switchable polymerization is an emerging method for synthesizing block copolymers in a one-pot, one-step process involving the reaction of multiple monomers with significantly different reactivities, which allows the polymerization reactions to switch automatically according to differences in reactivity [19,20,21,22,23,24]. For example, Williams and co-workers, pioneers in self-switchable polymerization, have synthesized poly(maleic anhydride-*alt*-propylene oxide)-*b*-poly(L-lactide) through the self-switchable polymerization of maleic anhydride, propylene oxide, and L-lactide (LLA), demonstrating its potential as an additive to enhance the properties of poly(L-lactide) (PLLA) [25]. On the other hand, our group has demonstrated a one-pot, one-step block copolymer synthesis from a mixture of cyclic anhydride, epoxide, and LLA using an alkali metal carboxylate as a catalyst [20]. By optimizing the soft segment properties by the judicious choice of cyclic anhydride and epoxide, we obtained PLLA-containing block copolymers that exhibited thermoplastic elastomer-like properties owing to the microphase separation between the soft segment and PLLA blocks. Thus, self-switchable polymerization based on ROAC has great potential in the design and development of novel sustainable polymer materials.

Hot-melt adhesives are widely used in industrial fields, and many rely on petroleum-based polymers such as vinyl polymers, necessitating a shift toward sustainable PEs. In this study, we aimed to develop a sustainable alternative to conventional hot-melt adhesives using a self-switchable polymerization approach. Thermoplastic elastomers with a “hard-soft-hard”-type triblock architecture, such as polystyrene-*b*-polyisoprene-*b*-polystyrene, have been widely used as hot-melt adhesives [26,27,28]. Based on similar concepts, hard PLLA segments attached to both ends of an ROAC-derived soft segment can function as an adhesive. Because of the bio-based and biodegradable nature of PLA, copolymers synthesized in this study represent a promising sustainable alternative for future adhesive applications.

**Scheme 1:**
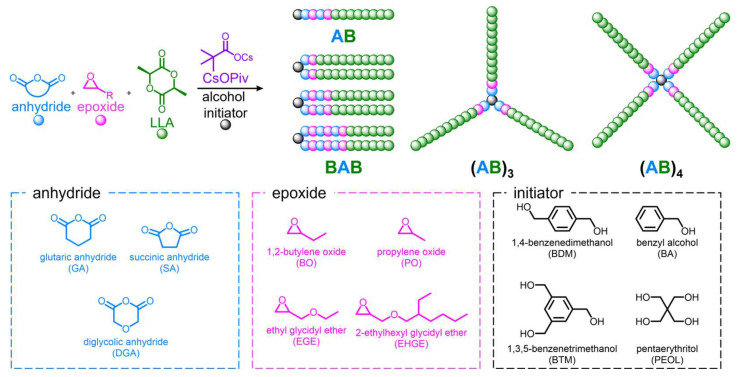
One-pot, one-step synthesis of polyester adhesives via self-switchable polymerization.

In this study, we utilized the self-switchable polymerization of cyclic anhydride, epoxide, and LLA to develop hot-melt adhesives with a high biomass content (Scheme 1). By changing the initiator, cyclic anhydride, and epoxide, we successfully synthesized a variety of copolyesters consisting of PLLA segments with various repeating structures, molecular weights, compositions, and architectures. Lap shear tests revealed that some of the synthesized copolyesters exhibited sufficient adhesive performance to a wooden substrate and could potentially substitute for commercial products such as PE-based and poly(vinyl acetate) (PVAc)-based hot-melt adhesives.

## 2. Experimental Section

### 2.1. Chemicals

L-Lactide (LLA; >98.0%, Tokyo Kasei Kogyo Co., Ltd. (TCI), Tokyo, Japan) was purified by recrystallizing it from dry toluene twice. 1,2-Butylene oxide (BO; >99.0%, TCI), 2-ethylhexyl glycidyl ether (EHGE; >98.0%, TCI), and ethyl glycidyl ether (EGE; >98.0%, TCI) were distilled over CaH_2_ under reduced pressure and stored under argon atmosphere. Propylene oxide (PO; >99.0%, TCI) was distilled over CaH_2_ and stored under argon atmosphere. Succinic anhydride (SA; >95.0%, TCI), diglycolic anhydride (DGA; >98.0%, TCI), and glutaric anhydride (GA; >98.0%, TCI) were purified by sublimation under reduced pressure. Cesium pivalate (CsOPiv; >97.0%, TCI) was dried by heating at 100 °C under high vacuum for at least 72 h before use. Benzyl alcohol (BA; >99.0%, TCI), 1,4-benzenedimethanol (BDM; >99.0%, TCI), 1,3,5-benzenetrimethanol (BTM; >95.0%, TCI), and pentaerythritol (PEOL; >98.0%, TCI) were used as received.

### 2.2. Instruments

Polymerization mixture was prepared in an MBRAUN stainless-steel glovebox equipped with a gas purification system (molecular sieves and copper catalyst) in a dry argon atmosphere (H_2_O, O_2_ < 0.1 ppm). The moisture and oxygen contents in the glovebox were monitored by MB-MO-SE-1 and MB-OX-SE-1, respectively.

^1^H, ^13^C, and DOSY NMR. The ^1^H (400 MHz) and ^13^C (100 MHz) NMR spectra were recorded using a JEOL JNM-ECS400 instrument (Tokyo, Japan). The diffusion-ordered NMR (DOSY) spectrum was recorded using a JEOL JNM-ECZ600R (600 MHz) instrument. DOSY analysis was carried out at 30 °C using the ledbpgp2s sequence with at least 15 gradient increments. 

Size exclusion chromatography (SEC). Size exclusion chromatography (SEC) was performed at 40 °C in THF (flow rate, 1.0 mL min^–1^) using a Jasco high-performance liquid chromatography system (PU-4180 HPLC pump, AS-4550 auto sampler, and CO-4060 column oven) equipped with a Shodex K-800D guard column (8.0 mm × 100 mm; particle size, 10 μm) and two Shodex columns (K-806L and K-804L; linear, 8.0 mm × 100 mm; particle size, 10 μm).

Differential scanning calorimetry (DSC). The DSC experiments were performed using a Hitachi High-Tech Science DSC 7000X under a nitrogen atmosphere. The samples were pre-heated by hot pressing them at 100 °C, following the same preparation as for the lap shear tensile test, and then aged at 10 °C for 7 days. After aging, the thin films were subjected to the DSC measurement, in which the sample was firstly cooled to −100 °C and heated to 180 °C at the cooling and heating rate of 10 °C min^−1^.

Thermogravimetric analysis (TGA). The TGA experiments were performed using a Hitachi High-Tech Science STA200RV under nitrogen atmosphere. The samples were heated from 30 °C to 600 °C at the heating rate of 10 °C min^−1^.

Lap shear adhesive test. The adhesive property was evaluated by lap shear adhesive tests. Four different kinds of adherents were used, including wood (Beech; 25 mm × 50 mm × 4 mm), aluminum (Al; 25 mm × 50 mm × 2 mm), iron (Fe; 25 mm × 50 mm × 1.6 mm), glass (25 mm × 50 mm × 2 mm), and poly(ethylene terephthalate) (25 mm × 50 mm × 0.1 mm). The samples for the lap shear test were obtained as follows: the polymer was hot pressed at 100 °C to produce 300 μm thick film, which were then cut into 12.5 mm × 25.0 mm. The cut films were sandwiched between the two adherents of the same kind, which were wiped using methanol to remove impurities before being used and pressed at 100 °C again. The sandwiched plates were kept at room temperature for seven days before tests. Lap shear adhesive test was carried out following JIS K 6850:1999 using an Instron 34SC-1 at room temperature. For each film, three samples were tested, and the average values along with their corresponding standard deviations were calculated.

### 2.3. Typical Synthesis of the Block Copolymers via Self-Switchable Polymerization

#### 2.3.1. Synthesis of PLLA-*tb*-poly(GA-*alt*-BO)-*tb*-PLLA (**P1**)

A typical polymerization procedure is given as follows: In an argon-filled glovebox, BDM (41.4 mg, 300 μmol), CsOPiv (70.2 mg, 300 μmol), GA (685 mg, 6.00 mmol), LLA (4.76 g, 33.0 mmol), and BO (1.73 g, 24.0 mmol) were added to the reaction vessel and sealed with a greaseless valve. After removing the vessel from the glovebox, the reaction mixture was stirred at 100 °C in an oil bath. After 5.5 h, crude aliquot was withdrawn from the system by pipette and monitored by ^1^H NMR spectroscopy in CDCl_3_ to determine the monomer conversions. The reaction mixture was diluted with small amount of dichloromethane to terminate the polymerization. The crude polymer was purified by reprecipitation in cold methanol to obtain poly(L-lactide)-*tapered block*-poly(glutaric anhydride-*alternating*-butylene oxide)-*tapered block*-poly(L-lactide) (PLLA-*tb*-poly(GA-*alt*-BO)-*tb*-PLLA) as a colorless solid (yield: 4.94 g, 83.5%).

*M*_n,NMR_ = 17,800 g mol^−1^ (CDCl_3_), *M*_n,SEC_ = 3990 g mol^−1^ (THF), *Đ* = 1.72 (THF)

#### 2.3.2. Synthesis of PLLA-*b*-poly(SA-*alt*-BO)-*b*-PLLA (**P6**)

PLLA-*block*-poly(SA-*alt*-BO)-*block*-PLLA (PLLA-*b*-poly(SA-*alt*-BO)-*b*-PLLA) was synthesized in the same way, except for the cyclic anhydride monomer and the molar ratios of the reactants, which were as follows: BDM (41.4 mg, 300 μmol), CsOPiv (70.2 mg, 300 μmol), SA (691 mg, 6.90 mmol), LLA (4.76 g, 33.0 mmol), and BO (1.99 g, 27.6 mmol). After 6 h, the reaction mixture was diluted with small amount of dichloromethane and purified by reprecipitation in cold methanol to PLLA-*b*-poly(SA-*alt*-BO)-*b*-PLLA as a black powder (yield: 5.77 g, 96.4%).

*M*_n,NMR_ = 20,000 g mol^−1^ (CDCl_3_), *M*_n,SEC_ = 8610 g mol^−1^ (THF), *Đ* = 1.46 (THF)

#### 2.3.3. Synthesis of PLLA-*b*-poly(DGA-*alt*-PO)-*b*-PLLA (**P7**)

PLLA-*b*-poly(DGA-*alt*-PO)-*b*-PLLA was synthesized in the same way, except for the cyclic anhydride monomer and the molar ratios of the reactants, which were as follows: BDM (20.7 mg, 150 μmol), CsOPiv (35.1 mg, 150 μmol), DGA (348 mg, 3.00 mmol), LLA (2.38 g, 16.5 mmol), and BO (865 mg, 12.0 mmol). After 3.5 h, the reaction mixture was diluted with small amount of dichloromethane and purified by reprecipitation in cold methanol to PLLA-*b*-poly(DGA-*alt*-BO)-*b*-PLLA as a colorless solid (yield: 2.80 g, 94.6%).

*M*_n,NMR_ = 23,700 g mol^−1^ (CDCl_3_), *M*_n,SEC_ = 11,200 g mol^−1^ (THF), *Đ* = 1.18 (THF)

#### 2.3.4. Synthesis of PLLA-*tb*-poly(GA-*alt*-PO)-*tb*-PLLA (**P8**)

PLLA-*tb*-poly(GA-*alt*-PO)-*tb*-PLLA was synthesized in the same way, except for the cyclic anhydride monomer and the molar ratios of the reactants, which were as follows: BDM (27.6 mg, 200 μmol), CsOPiv (46.8 mg, 200 μmol), GA (571 mg, 5.00 mmol), LLA (3.17 g, 22.0 mmol), and PO (1.16 g, 20.0 mmol). After 5 h, the reaction mixture was diluted with small amount of dichloromethane and purified by reprecipitation in cold methanol to PLLA-*tb*-poly(GA-*alt*-PO)-*tb*-PLLA as a colorless solid (yield: 3.72 g, 91.6%).

*M*_n,NMR_ = 22,800 g mol^−1^ (CDCl_3_), *M*_n,SEC_ = 5380 g mol^−1^ (THF), *Đ* = 1.51 (THF)

#### 2.3.5. Synthesis of PLLA-*tb*-poly(GA-*alt*-EGE)-*tb*-PLLA (**P9**)

PLLA-*tb*-poly(GA-*alt*-EGE)-*tb*-PLLA was synthesized in the same way, except for the epoxide monomer and the molar ratio of the reactants, which were as follows: BDM (27.6 mg, 200 μmol), CsOPiv (46.8 mg, 200 μmol), GA (456 mg, 4.00 mmol), LLA (3.17 g, 22.0 mmol), and EGE (1.63 g, 16.0 mmol). After 4 h, the reaction mixture was diluted with small amount of dichloromethane and purified by reprecipitation in cold methanol to PLLA-*tb*-poly(GA-*alt*-EGE)-*tb*-PLLA as a colorless solid (yield: 4.20 g, 98.2%).

*M*_n,NMR_ = 24,700 g mol^−1^ (CDCl_3_), *M*_n,SEC_ = 4160 g mol^−1^ (THF), *Đ* = 1.84 (THF)

#### 2.3.6. Synthesis of PLLA-*tb*-poly(GA-*alt*-EHGE)-*tb*-PLLA (**P10**)

PLLA-*tb*-poly(GA-*alt*-EHGE)-*tb*-PLLA was synthesized in the same way, except for the epoxide monomer and the molar ratios of the reactants, which were as follows: BDM (27.6 mg, 200 μmol), CsOPiv (46.8 mg, 200 μmol), GA (342 mg, 3.00 mmol), LLA (3.46 g, 24.0 mmol), and EHGE (2.24 g, 24.0 mmol). After 6.5 h, the reaction mixture was diluted with small amount of dichloromethane and purified by reprecipitation in cold methanol to PLLA-*tb*-poly(GA-*alt*-EHGE)-*tb*-PLLA as a colorless solid (yield: 3.80 g, 86.6%).

*M*_n,NMR_ = 23,000 g mol^−1^ (CDCl_3_), *M*_n,SEC_ = 8260 g mol^−1^ (THF), *Đ* = 1.39 (THF)

#### 2.3.7. Synthesis of AB-type poly(GA-*alt*-BO)-*tb*-PLLA (**P11**)

poly(GA-*alt*-BO)-*tb*-PLLA was synthesized in the same way, except for the initiator and the molar ratios of the reactants, which were as follows: BA (32.4 mg, 300 μmol), CsOPiv (70.2 mg, 300 μmol), GA (411 mg, 3.60 mmol), LLA (2.59 g, 18.0 mmol), and BO (1.04 g, 14.4 mmol). After 5.5 h, the reaction mixture was diluted with small amount of dichloromethane and purified by reprecipitation in cold methanol to poly(GA-*alt*-BO)-*tb*-PLLA as a colorless solid (yield: 2.23 g, 67.6%).

*M*_n,NMR_ = 10,800 g mol^−1^ (CDCl_3_), *M*_n,SEC_ = 5060 g mol^−1^ (THF), *Đ* = 1.59 (THF)

#### 2.3.8. Synthesis of (poly(GA-*alt*-BO)-*tb*-PLLA)_3_ (**P12**)

(poly(GA-*alt*-BO)-*tb*-PLLA)_3_ was synthesized in the same way, except for the initiator and the molar ratios of the reactants, which were as follows: BTM (16.8 mg, 100 μmol), CsOPiv (23.4 mg, 100 μmol), GA (399 mg, 3.50 mmol), LLA (2.45 g, 17.0 mmol), and BO (1.01 g, 14.0 mmol). After 8.0 h, the reaction mixture was diluted with small amount of dichloromethane and purified by reprecipitation in cold methanol to (poly(GA-*alt*-BO)-*tb*-PLLA)_3_ as a colorless solid (yield: 2.61 g, 83.8%).

*M*_n,NMR_ = 32,600 (CDCl_3_), *M*_n,SEC_ = 8110 g mol^−1^ (THF), *Đ* = 1.22 (THF)

#### 2.3.9. Synthesis of PLLA-*tb*-poly(GA-*alt*-BO)-*tb*-PLLA (10k) (**P13**)

PLLA-*tb*-poly(GA-*alt*-BO)-*tb*-PLLA (10k) was synthesized in the same way, except for the molar ratios of the reactants, which were as follows: BDM (69.1 mg, 500 μmol), CsOPiv (117 mg, 500 μmol), GA (571 mg, 5.00 mmol), LLA (3.96 g, 27.5 mmol), and BO (1.44 g, 20.0 mmol). After 4 h, the reaction mixture was diluted with small amount of dichloromethane and purified by reprecipitation in cold methanol to PLLA-*tb*-poly(GA-*alt*-BO)-*tb*-PLLA (10k) as a colorless solid (yield: 4.96 g, 99.8%).

*M*_n,NMR_ = 11,800 g mol^−1^ (CDCl_3_), *M*_n,SEC_ = 2180 g mol^−1^ (THF), *Đ* = 2.12 (THF)

#### 2.3.10. Synthesis of PLLA-*tb*-poly(GA-*alt*-BO)-*tb*-PLLA (40k) (**P14**)

PLLA-*tb*-poly(GA-*alt*-BO)-*tb*-PLLA (40k) was synthesized in the same way, except for the molar ratios of the reactants, which were as follows: BDM (27.6 mg, 200 μmol), CsOPiv (46.8 mg, 200 μmol), GA (958 mg, 8.40 mmol), LLA (6.34 g, 44.0 mmol), and BO (2.42 g, 33.6 mmol). After 9 h, the reaction mixture was diluted with small amount of dichloromethane and purified by reprecipitation in cold methanol to PLLA-*tb*-poly(GA-*alt*-BO)-*tb*-PLLA (40k) as a colorless solid (yield: 7.52 g, 94.7%).

*M*_n,NMR_ = 38,100 g mol^−1^ (CDCl_3_), *M*_n,SEC_ = 6510 g mol^−1^ (THF), *Đ* = 1.53 (THF)

#### 2.3.11. Synthesis of (poly(GA-*alt*-BO)-*tb*-PLLA)_4_ (80k) (**P15**)

(poly(GA-*alt*-BO)-*tb*-PLLA)_4_ (80k) was synthesized in the same way, except for the initiator and the molar ratios of the reactant, which were as follows: PET (9.5 mg, 70 μmol), CsOPiv (16.4 mg, 70 μmol), GA (671 mg, 5.88 mmol), LLA (4.44 g, 30.8 mmol), and BO (1.70 g, 23.5 mmol). After 23 h, the reaction mixture was diluted with small amount of dichloromethane and purified by reprecipitation in cold methanol to (poly(GA-*alt*-BO)-*tb*-PLLA)_4_ (80k) as a colorless solid (yield: 4.96 g, 89.4%).

*M*_n,NMR_ = *N.D.*, *M*_n,SEC_ = 9170 g mol^−1^ (THF), *Đ* = 1.62 (THF)

## 3. Results

### 3.1. Self-Switchable Polymerization Using GA, BO, and LLA

**Figure 1:**
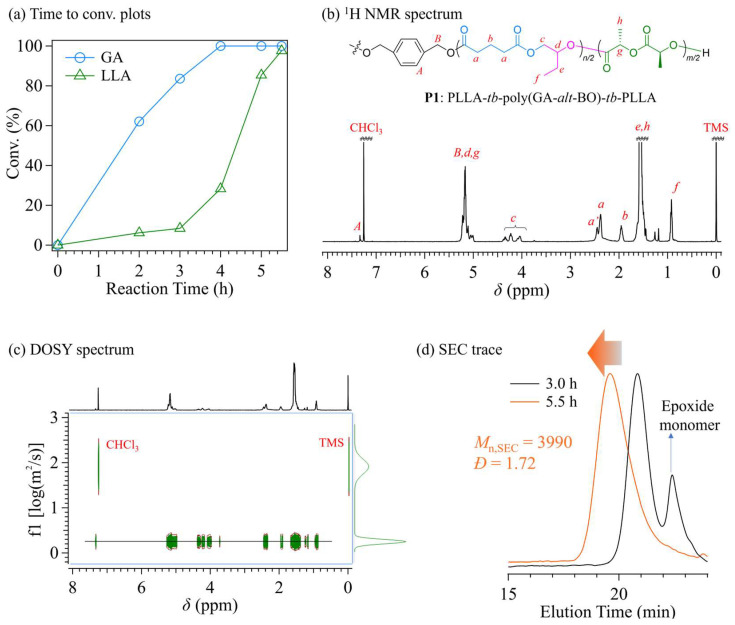
Characterization of **P1**. (**a**) Time to conv. plots monitored by ^1^H NMR. (**b**) ^1^H NMR and (**c**) DOSY spectrum in CDCl_3_. (**d**) SEC traces of the product withdrawn at 3.0 h and the final product (5.5 h) (eluent, THF; flow rate, 1.0 mL min^−1^). Signal *a′* of ^1^H NMR corresponding to the proton signal from the GA unit adjacent to the LLA unit.

We initially selected glutaric anhydride (GA), 1,2-butylene oxide (BO), and LLA to explore the optimized conditions for the one-pot, one-step synthesis of “hard-soft-hard”-type triblock copolymers via self-switchable polymerization. In addition to LLA, GA and BO can potentially be produced from bio-based raw materials; therefore, they are considered as potential bio-based monomers [29,30]. In our previous study, we found that the ROAC of cyclic anhydride and epoxide was more reactive than the ring-opening polymerization (ROP) of LLA in cesium-pivalate-catalyzed self-switchable polymerization using cyclic anhydride, epoxide, and LLA. Consequently, the terpolymerization of the cyclic anhydride, epoxide, and LLA in the presence of a diol is expected to yield a hard-soft-hard triblock copolymer, that is, PLLA-*b*-poly(GA-*alt*-BO)-*b*-PLLA. First, polymerization was attempted with an initial feed ratio [CsOPiv]/[BDM]_0_/[GA]_0_/[BO]_0_/[LLA]_0_ of 1/1/20/80/110 to obtain a copolyester consisting of an LLA weight fraction of 0.80 (**P1** in Table 1). The time course of the copolymerization was monitored via ^1^H NMR to gain insight into the monomer sequence of the obtained copolyester. The time-to-conversion plots of GA and LLA revealed that the ROAC of GA and BO proceeded preferentially, while the ROP of LLA also proceeded very slowly at the same time, owing to the similar reactivity of GA and LLA (Figure 1a). Thus, the obtained copolyester was found to possess a large tapered region with a poly(GA-*alt*-BO)-rich structure in the middle and a PLLA-rich structure in the outer parts, that is, PLLA-*tapered block*-poly(GA-*alt*-BO)-*tapered block*-PLLA (PLLA-*tb*-poly(GA-*alt*-BO)-*tb*-PLLA). **P1** was characterized using ^1^H NMR and size exclusion chromatography (SEC) measurements. The ^1^H NMR spectrum of **P1** revealed signals corresponding to the initiator residue (–CH_2_C_6_*H_4_*CH_2_- (*A*), 7.34 ppm), poly(GA-*alt*-BO) repeating unit (-OCOC*H_2_*CH_2_C*H_2_*COO- (*a*), 2.24–2.56 ppm; -OCOCH_2_C*H_2_*CH_2_COO- (*b*), 1.83–2.06 ppm; -OC*H_2_*CH(CH_2_CH_3_)O- (*c*), 3.91–4.49 ppm; -OCH_2_C*H*(CH_2_CH_3_)O- (*d*), 4.90–5.05 ppm; –C*H_2_*CH_3_ (*e*), 1.34–1.81 ppm; –CH_2_C*H_3_* (*f*), 0.70–0.99 ppm), and PLLA repeating unit (–C*H*CH_3_ (*g*), 5.05–5.28 ppm; –CHC*H_3_* (*h*), 1.34–1.81 ppm), as expected (Figure 1b). Additionally, the LLA weight fraction determined from the ^1^H NMR spectrum (*F*_LLA_ = 0.83) closely matches that calculated from the monomer feed ratio (*f*_LLA_ = 0.81). We noted the signal *a′* originating from the GA unit proton adjacent to the LLA unit was observed at 2.4 ppm, supporting the presence of the tapered region (Figure S1). In addition, the ^13^C NMR spectrum showed the signal *a′*, indicating a tapered region (Figure S2). The diffusion-ordered NMR (DOSY) spectra provided only one diffusion coefficient for the observed signals of both poly(GA-*alt*-BO) and PLLA, providing strong evidence that these segments were covalently linked (Figure 1c). The SEC traces maintained a unimodal shape while shifting toward higher molecular weights over time, confirming that both segments were interconnected (Figure 1d). The calculated polystyrene-equivalent number average molecular weight (*M*_n,SEC_) and polydispersity (*Ð*) were 3990 and 1.72, respectively. These results demonstrate the one-pot and one-step synthesis of BAB-type tapered-block copolyester with A and B blocks of poly(GA-*alt*-BO) and PLLA, respectively. In the same manner, the copolyesters P2–P5, which consist of *F*_LLA_ ranging from 0.25 to 0.87, were synthesized by changing *f*_LLA_ (Table 1, Figures S3–S7).

### 3.2. Thermal Properties of PLLA-tb-poly(GA-alt-BO)-tb-PLLA

**Figure 2:**
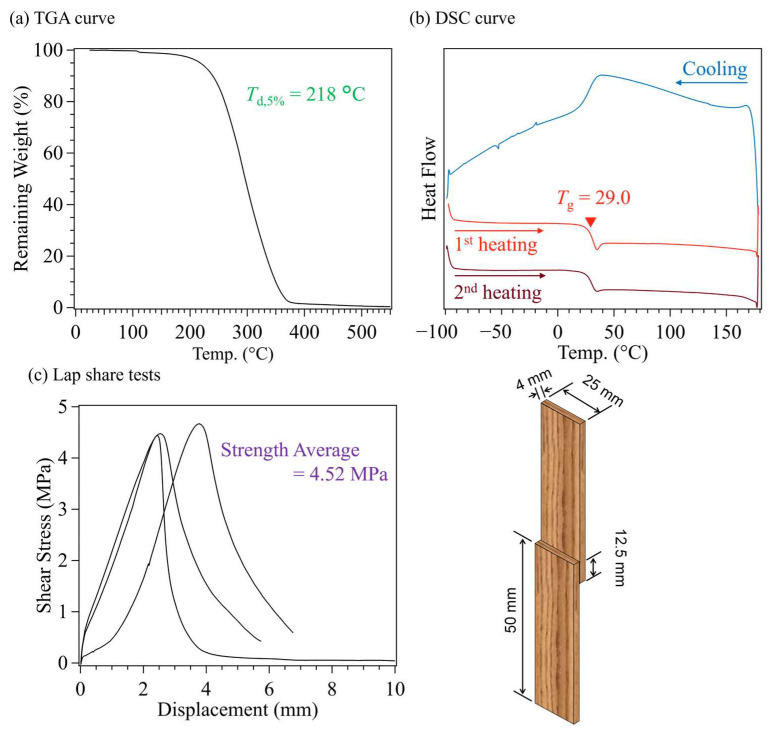
Physical properties of **P1**: (**a**) TGA curve (heating ratio, 10 °C min^−1^; nitrogen atmosphere). (**b**) DSC thermogram detected during heating/cooling/heating scan (1st heating: −100–180 °C, 1st cooling: −100–180 °C, 2nd heating: −100–180 °C; 10 °C min^−1^; N_2_ atmosphere). (**c**) Lap share tests using wood pieces (adherent, wood; tensile speed = 10 mm min^−1^; *n* = 3).

The thermal properties of **P1**–**P5** were investigated via a thermogravimetric analysis (TGA) and differential scanning calorimetry (DSC) under a N_2_ atmosphere. The TGA revealed a 5% weight loss temperature (*T*_d,5%_) of 189–244 °C, implying that these copolyesters were useful below 180 °C (Figure 2a and Figure S8).

DSC measurements of **P1** were performed at a heating and cooling rate of 10 °C (Figure 2b). Neither crystallization nor melting transitions were observed during the first heating process, implying that the crystallization of PLLA was inhibited by the presence of the poly(GA-*alt*-BO) repeating units in the PLLA segment. Another reason for poor crystallization in the PLLA segment could be partial epimerization (see Figure S2). In the ^13^C NMR spectrum of **P1**, the signal corresponding to the carbonyl carbons (signal *a*) around 169.5 ppm shows splitting, indicating that epimerization occurred. **P1** exhibited a glass transition temperature (*T*_g_) of 29.0 °C, which is in between the *T*_g_ of PLLA (60 °C) [31] and poly(GA-*alt*-BO) (−43.1 °C), suggesting that the two segments are miscible. The *T*_g_ was calculated using the Fox equation (*T*_g,calc._ = 35.7 °C) and is in good agreement with the measured *T*_g_ of 29.0 °C, supporting the miscibility of the two segments. Similarly, only one *T*_g_ was observed for **P2**–**P5**, and their *T*_g_ values agreed with *T*_g,calc._ (Figure S9, Table S1). Based on the DSC results, we confirmed that PLLA-*tb*-poly(GA-*alt*-BO)-*tb*-PLLAs were amorphous and exhibited *T*_g_ values ranging from −15.6 to 32.5 °C depending on *F*_LLA_. The thermal properties of the synthesized copolymers are worse than those of commercial adhesives. However, improvements are anticipated through optimized monomer selection and other refinement strategies in the future.

### 3.3. Evaluation of Adhesive Properties Using Lap Shear Test

Next, we investigated the adhesive properties of the obtained PLLA-*tb*-poly(GA-*alt*-BO)-*tb*-PLLAs by lap shear tests using wood test pieces at room temperature (JIS K 6850:1999; the right side in Figure 2c and Figure S10). Test samples were prepared by pressing polymer thin film between two wooden substrates at 100 °C.

**Figure 3:**
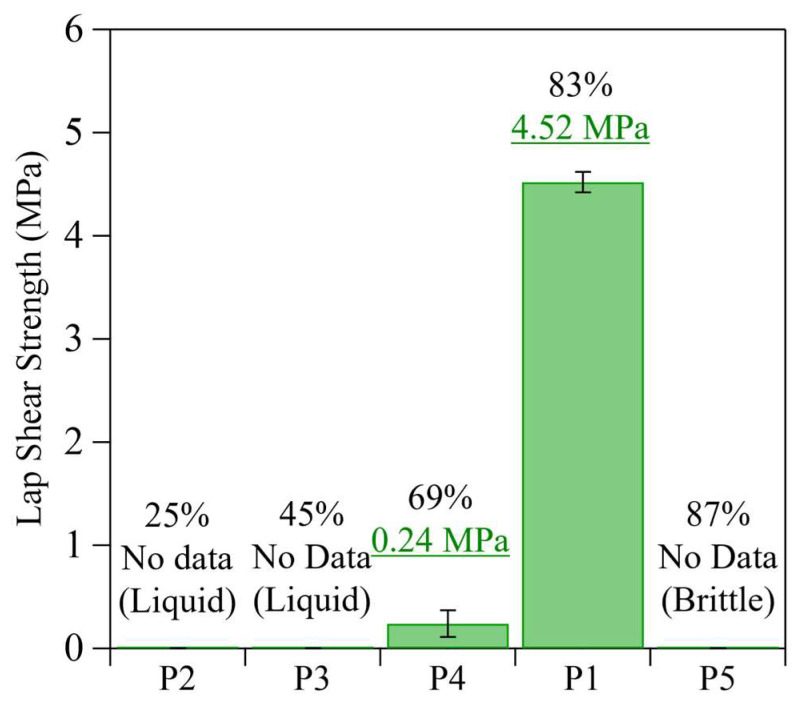
Lap shear strengths of PLLA-*tb*-poly(GA-*alt*-BO)-*tb*-PLLA with varying compositions in *F*_LLA_ (adherent, wood; tensile speed = 10 mm min^−1^; *n* = 3).

We were unable to prepare the test samples from **P2**, **P3**, and **P5** (*F*_LLA_ = 0.25, 0.45, and 0.87, respectively) as well as poly(GA-*alt*-BO) and PLLA homopolymers because of the following reasons (Figure S11): For **P2**, **P3**, and poly(GA-*alt*-BO), we could not obtain a self-standing film for the hot-melt adhesive application due to their low *T*_g_ as well as their liquid nature (their appearance was viscous liquid even at 10 °C). In contrast, **P5** and PLLA are brittle solids; therefore, effective adhesion cannot be achieved. In contrast, we successfully prepared a self-standing film from **P1** and **P4** that functioned as a hot-melt adhesive. A lap shear test on the **P1** sample (Figure 2c) revealed an adhesive strength of 4.52 MPa, indicating cohesion failure. While **P1** demonstrated a high adhesive strength to the wooden substrate, **P4**, with an *F*_LLA_ of 0.69, showed a much lower strength (0.24 MPa (Figure 3 and Figure S12)). Although the adhesive strength appears to increase with the increasing *F*_LLA_, pure PLLA is too brittle to function as an adhesive. This indicates the importance of combining the PLLA and poly(GA-*alt*-BO) blocks in a balanced composition to achieve sufficient adhesive performance.

To confirm the importance of chemically linking PLLA and poly(GA-*alt*-BO) blocks for adhesive performance, a polymer blend with a composition corresponding to **P1** was prepared by mixing the two homopolymers (PLLA/poly(GA-*alt*-BO) = 83/17 (weight fraction)) at 100 °C for 2 min, following hot pressing at 100 °C for 1 min (Table S2). As shown in Figure S13, the DSC curve of the PLLA/poly(GA-*alt*-BO) blend sample shows only one *T*_g_, suggesting that these two polymers have a high level of miscibility. The adhesive strength of the blended sample to the wooden substrate was only one-third that of **P1** (1.20 MPa, Figures S14 and S15). Thus, we successfully demonstrated that copolymerization, rather than two-homopolymer blending, is a significant contributor to adhesive performance.

### 3.4. Effect of Monomer Combinations 

To evaluate the effect of the repeating structure of the poly(cyclic anhydride-*alt*-epoxide) segment, we then prepared **P6**–**P10** using several anhydrides and epoxides (Table 1). This study aimed to synthesize a simple aliphatic polyester copolymer, and we thus employed commercially available and easily accessible aliphatic monomers. First, we carried out self-switchable polymerization under optimized conditions with a BDM initiator using succinic anhydride (SA) and diglycolic anhydride (DGA) as the anhydride monomers, while fixing BO as the epoxide monomer (**P6**–**P7** in Table 1). Here, we set the *f*_LLA_ to approximately 0.80 because the lap shear results in the previous section indicate that an *F*_LLA_ of 0.83 exhibits excellent adhesive properties. The SEC traces of the final products showed a unimodal peak, confirming successful copolymerization (Figure S16). Monitoring the progress of the polymerization by ^1^H NMR revealed the switching of the polymerization from the ROAC of anhydrides and BO to the ROP of LLA, suggesting the formation of a BAB-type sequence with PLLA as the B block (Figure S17). Owing to the insufficient sampling frequency, the exact switching timing could not be determined. However, the signals assignable to the LLA-(anhydride-*alt*-BO) and (anhydride-*alt*-BO)-LLA units were almost undetectable in the ^13^C NMR spectra of **P6** and **P7**, suggesting a real block copolymer structure rather than a tapered structure, that is, PLLA-*b*-poly(SA-*alt*-BO)-*b*-PLLA and PLLA-*b*-poly(DGA-*alt*-BO)-*b*-PLLA (Figures S18–S21). This is due to the much higher reactivities of SA and DGA compared to that of LLA, which enabled faster switching from ROAC to ROP. The DSC curves of **P6** and **P7** show no crystallization or melting peaks, suggesting their amorphous nature (Figure S22). Although the observed *T*_g_ is almost the same as **P1**, which consisted of GA, the **P6** and **P7** samples were too brittle, and the preparation of adhesive test samples was unsuccessful. 

Next, to synthesize copolyesters with different epoxide moieties, we carried out the self-switchable copolymerization of GA and LLA with several different epoxides, i.e., propylene oxide (PO), ethyl glycidyl ether (EGE), and 2-ethylhexyl glycidyl ether (EHGE) (**P8**–**P10** in Table 1). The ROP of LLA proceeded slowly, while the ROAC of GA and epoxides proceeded, as evidenced by reaction monitoring by ^1^H NMR, indicating that the obtained copolymers possessed a significantly tapered region (Figure S23). The ^1^H NMR and SEC analyses revealed an *F*_LLA_ of 0.78–0.88 and a narrow-to-moderate *Ð* of 1.39–1.84, respectively (Figures S24–S29). The *T*_g_ values of **P8**–**P10** were observed at 1.3–15.1 °C from the DSC curves (Figure S22). The copolyesters synthesized using glycidyl ethers (**P9** and **P10**) were found to show particularly low *T*_g_ (ca. 1 °C) values, which could be due to the flexibility of the glycidyl ether side chain. Lap shear samples are difficult to prepare because of their low *T*_g_. The adhesive strengths of **P8**–**P10** are shown in Figure S30. Compared to the adhesive strengths of **P9** and **P10** (0.05 MPa and 0.56 MPa), **P1** and **P8** showed a much higher adhesive strength (4.52 MPa and 2.49 MPa; Figure S31). This trend seems to correlate with the *T*_g_ of the copolyesters, in which alkylene oxide monomers lead to higher *T*_g_ values than glycidyl ethers. Overall, we found that altering the epoxide monomer was an effective means of adjusting the adhesion performance. Because altering the cyclic anhydride significantly affects the main chain structure as well as the monomer sequence, this results in substantial effects on the adhesive properties. In contrast, altering the epoxide monomer only affected the side chains. Because the side chain structure allows for the precise control of *T*_g_, modifying the epoxide monomer is effective in tuning the adhesive properties.

### 3.5. Effect of Branched Structure and Molecular Weight on Adhesive Properties

To further improve the adhesive properties, we investigated the effects of the polymer chain architecture and molecular weight. To reveal these relationships, we synthesized poly(GA-*alt*-BO)-*tb*-PLLA with different branching structures and total molecular weights (**P11**–**P15** in Table 2). Linear AB diblock and three-armed (AB)_3_ star block-type copolyesters were synthesized by the self-switchable polymerization of GA, BO, and LLA under established conditions while altering the initiator from BDM to benzyl alcohol (BA) and 1,3,5-benzenetrimethanol (BTM) (**P11** and **P12**). In the ^1^H NMR spectrum of **P11**, signal *A* corresponding to the initiator moiety was clearly observed at 7.35 ppm (Figure S32). The ^1^H NMR spectrum of **P12** also displayed the proton signals representing the initiator residue ((*A*), 7.25 ppm; (*B*), 5.16 ppm in Figure S33). The SEC traces of **P11** and **P12** showed a unimodal elution peak (**P11**: *M*_n,SEC_ = 5060, *Đ* = 1.59; **P13**: *M*_n,SEC_ = 8110, *Đ* = 1.22; Figures S25 and S26), and the ^1^H NMR revealed *F*_LLA_ values of 0.80 and 0.76, respectively. The adhesive strength values of the obtained **P11** and **P12** were 3.41 and 5.20 MPa (Figure S34), and **P1** with the BAB architecture is located in between **P11** and **P12**. These results suggest that the adhesive strength increased with an increasing number of arms.

Next, to evaluate the effect of the total molecular weight on the adhesive properties, copolyesters with a comparable *f*_LLA_ to **P1** but with smaller and larger molecular weights were synthesized by changing the initial [monomer]_0_/[initiator]_0_ ratios (**P13** and **P14** in Table 2). The *M*_n,NMR_ spectra of the obtained products are similar to those of *M*_n,theo._ (**P13**: *M*_n,theo._ = 9930, *M*_n,NMR_ = 11,800; **P14**: *M*_n,theo._ = 39,700; *M*_n,NMR_ = 38,100), and the *F*_LLA_ ratio was determined to be 0.78–0.80. In addition, the *T*_g_ values of **P13** and **P14** were evaluated to be 21.0 and 25.0 °C, which are comparable to that of **P1** (*T*_g_ = 19.0 °C). The adhesive strength values of **P13** and **P14** were determined to be 0.06 and 6.31 MPa, respectively (Figure S35). When compared with the results of **P1** with a compatible *F*_LLA_, it was demonstrated that the adhesive strength could be improved by increasing the molecular weight. This is likely due to the increased molecular weight, which leads to a greater entanglement of the polymer chains. From these investigations, increasing the molecular weight and number of branches while fixing the *F*_LLA_ at approximately 0.80 was found to be an effective strategy for improving the adhesive properties.

**Figure 4:**
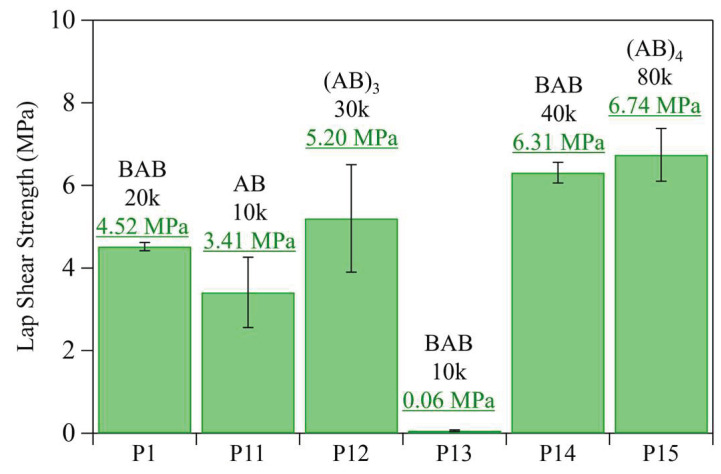
Lap shear strengths of poly(GA-*alt*-BO)-*tb*-PLLA copolyesters with different molecular weights and branched structures (adherent, wood; tensile speed = 10 mm min^−1^; *n* = 3).

**Figure 5:**
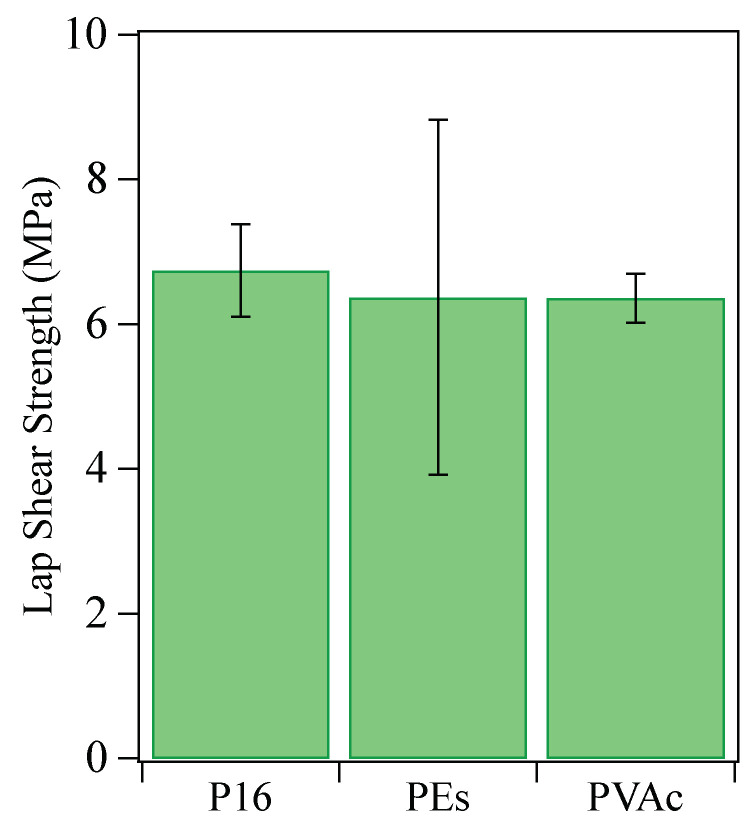
Lap shear strength of **P15** compared to commercial polyester (PE; PES-111, Toagosei) and poly(vinyl acetate) (PVAc; ref. [32]) adhesives (adhesion, wood; tensile speed = 10 mm min^−1^; *n* = 3).

Based on these results, a four-armed (AB)_4_-type copolyester with the highest molecular weight (*M*_n,theo._ of 79,200) and *F*_LLA_ of 0.79 was synthesized using a tetraol, i.e., pentaerythritol (PEOL), as an initiator with the initial feed ratio of [CsOPiv]/[PEOL]_0_/[GA]_0_/[BO]_0_/[LLA]_0_ = 1/1/84/336/440 (**P15** in Table 2). As expected, the adhesive strength of **P15** (6.74 MPa) was the highest among all the tested samples (Figure 4). Importantly, the adhesive strength of **P15** was comparable to that of commercially available PE and PVAc adhesives [29] (Figure 5).

**Figure 6:**
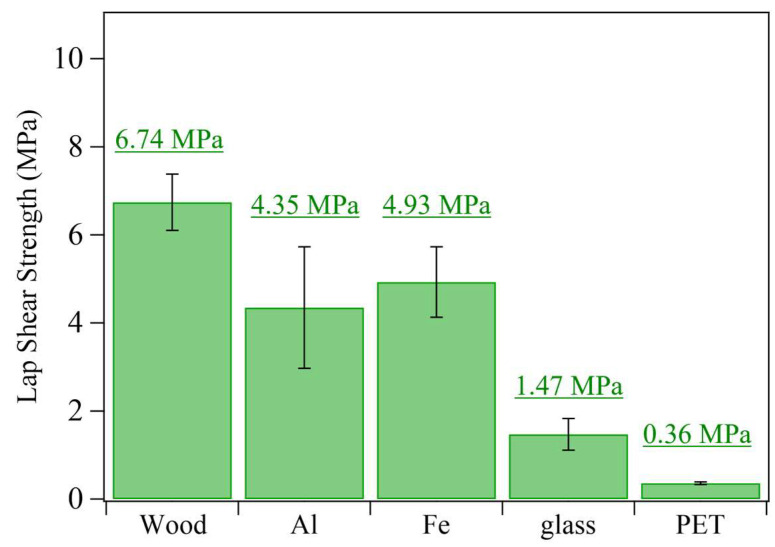
Lap shear strengths of **P15** on different adherents (tensile speed = 10 mm min^−1^; *n* = 3).

**Figure 7:**
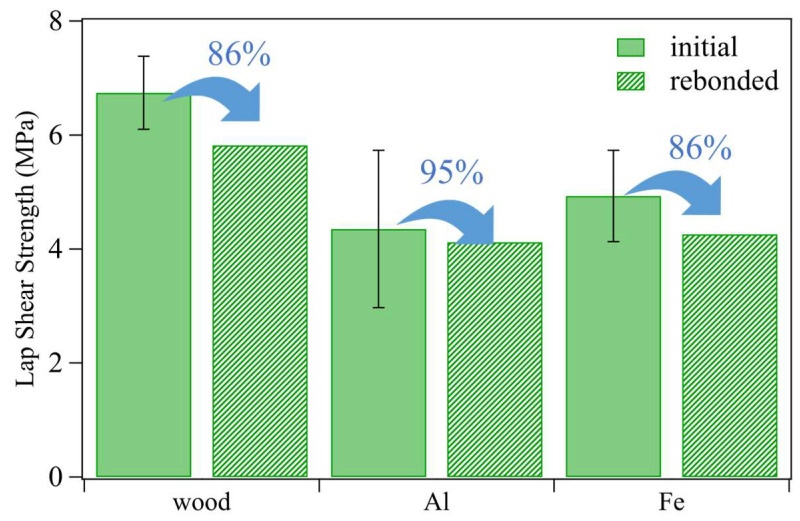
Lap shear strength of **P15** adhered wood, Al, and Fe pieces for both initial (*n* = 3) and rebounded (*n* = 1) samples. The test was conducted at a tensile speed of 10 mm min^−1^.

Using **P15**, we investigated the substrate versatility and recyclability of the copolyester adhesives. In addition to the wooden substrate, an aluminum plate (Al), iron plate (Fe), glass plate (glass), and poly(ethylene terephthalate) (PET) film were used as adherents (Figure 6). **P15** was found to function as a good adhesive for these substrates, except for PET. Finally, we conducted a recycling test on the **P15** adhesive (Figure 7 and Figures S36–S38). Following the lap shear adhesive test, the fractured samples were subjected to hot pressing at 100 °C for 1 min and cured for 1 week to generate recycled samples. Adhesive tests of the recycled samples revealed adhesive resilience values of 86%, 95%, and 86% for the wood, aluminum, and iron substrates, respectively, demonstrating sufficient recyclability. 

## 4. Conclusions

In this study, we succeeded in the one-pot, one-step synthesis of PE-based hot-melt adhesives with a high bio-based content using a self-switchable polymerization approach. The copolyesters consisting of poly(cyclic anhydride-*alt*-epoxide) and PLLA functioned as hot-melt adhesives. Taking advantage of the ease of self-switchable polymerization, a series of copolyesters with different molecular weights, PLLA weight fractions, repeating unit structures, and branched structures were readily prepared and subjected to lap shear tests, providing molecular design guidance for high-performance adhesives. Because these adhesives can adhere not only to wood, but also to glass, Fe, and Al substrates, they can be applied in a wide range of industrial fields. Moreover, the bio-based and biodegradable properties of the newly developed copolymer adhesives present significant potential for their use with bio-based and biodegradable products, such as biodegradable plastics, wood products, and paper. In this study, self-switchable polymerization was demonstrated as a powerful tool for the efficient exploration of new sustainable polymer materials. This strategy would be useful not only for hot-melt adhesives but also for the future development of tough plastics and elastomers with high bio-based contents and excellent biodegradability.

## Figures and Tables

**Table 1:** Molecular characteristics of copolyesters consisting of PLLA and different poly(cyclic anhydride-*alt*-epoxide) *^a^*.

Sample	Monomer Combination	[CsOPiv]/[BDM]_0_/ [anhydride]_0_/ [epoxide]_0_/[LLA]_0_	*f*_LLA_ *^b^*	Time (h)	*M*_n,theo._ *^c^*	*M*_n,NMR_ *^d^*	*M*_n,sec_ [*Ð*]* ^e^*	*F*_LLA_ * ^f^*	*T*_g_ *^g^*	*T*_d,5%_ *^h^*
**P1**	GA/BO/ LLA	1/1/20/80/110	0.81	5.5	19,700	17,800	3990 [1.72]	0.83	19.0	218
**P2**	GA/BO/ LLA	1/1/65/260/55	0.40	13.5	20,200	14,800	9550 [1.20]	0.25	−15.6	244
**P3**	GA/BO/ LLA	1/1/45/180/85	0.59	7.0	20,800	18,300	8090 [1.30]	0.45	−6.1	189
**P4**	GA/BO/ LLA	1/1/36/144/110	0.70	6.0	22,700	28,200	5200 [1.57]	0.69	13.7	196
**P5**	GA/BO/ LLA	1/1/11/44/130	0.90	4.5	20,900	23,500	6420 [1.36]	0.87	32.5	184
**P6**	SA/BO/ LLA	1/1/23/92/110	0.80	6.0	20,000	20,000	8610 [1.46]	0.81	31.4	207
**P7**	DGA/BO/ LLA	1/1/20/80/110	0.81	3.5	19,800	23,700	11,200 [1.18]	0.80	34.9	216
**P8**	GA/PO/ LLA	1/1/25/100/110	0.79	5.0	20,300	22,800	5380 [1.51]	0.74	15.7	194
**P9**	GA/EGE/ LLA	1/1/20/80/110	0.79	4.0	20,300	24,700	4160 [1.84]	0.77	1.4	188
**P10**	GA/EHGE/ LLA	1/1/15/60/120	0.79	6.5	21,900	23,000	8260 [1.39]	0.77	1.3	210

*^a^* Polymerization conditions: temp., 100 °C; atmosphere, Ar; initiator, BDM; catalyst, CsOPiv. *^b^* LLA weight fraction calculated from the monomer feed ratio. *^c^* Calculated from (M.W. of BDM) + [anhydride]_0_/[BDM]_0_ × (M.W. of anhydride + M.W. of epoxide) + [LLA]_0_/[BDM]_0_ × (M.W. of LLA). *^d^* Determined by ^1^H NMR spectroscopy in CDCl_3_. *^e^* Determined by SEC in THF using polystyrene standards. *^f^* LLA weight fraction calculated from ^1^H NMR spectrum. *^g^* Determined by DSC (first heating run, 10 °C min^−1^, nitrogen atmosphere). *^h^* Determined using TGA (10 °C min^−1^, nitrogen atmosphere).

**Table 2:** Molecular characteristics of copolyesters with different branched structures and molecular weights *^a^*.

Sample	Arm	Ini.	[CsOPiv]/ [Initiator]_0_/[GA]_0_/ [BO]_0_/[LLA]_0_	*f*_LLA_ *^b^*	Time (h)	*M*_n,theo._ *^c^*	*M*_n,NMR_ *^d^*	*M*_n,sec_ [*Ð*] *^e^*	*F*_LLA_ *^f^*	*T*_g_ *^g^*	*T*_d,5%_ *^h^*
**P11**	1	BA	1/1/12/48/60	0.80	5.5	11,000	10,800	5060 [1.59]	0.80	23.2	254
**P12**	3	BTM	1/1/35/140/170	0.79	8.0	31,200	32,600	8110 [1.22]	0.76	23.2	226
**P13**	2	BDM	1/1/10/40/55	0.81	4.0	9930	11,800	2180 [2.12]	0.78	21.0	190
**P14**	2	BDM	1/1/42/168/220	0.80	9.0	39,700	38,100	6510 [1.69]	0.80	25.0	224
**P15**	4	PEOL	1/1/84/336/440	0.80	23.0	79,200	N.D.	9170 [1.62]	0.79	21.2	235

*^a^* Polymerization conditions: temp., 100 °C; atmosphere, Ar; initiator, BDM; catalyst, CsOPiv. *^b^* LLA weight fraction calculated from the monomer feed ratio. *^c^* Calculated as (M.W. of initiator) + [GA]_0_/[initiator]_0_ × (M.W. of GA + M.W. of BO) + [LLA]_0_/[initiator]_0_ × (M.W. of LLA). *^d^* Determined by ^1^H NMR spectroscopy in CDCl_3_. *^e^* Determined by SEC in THF using polystyrene standards. *^f^* LLA weight fraction calculated from ^1^H NMR spectrum. *^g^* Determined by DSC (first heating run, 10 °C min^−1^, nitrogen atmosphere). *^h^* Determined using TGA (10 °C min^−1^, nitrogen atmosphere).

## Data Availability

The data presented in this study are available upon request from the corresponding author.

## Supplementary Materials

The following supporting information can be downloaded at: https://www.mdpi.com/article/10.3390/polym16192767/s1, Supplementary File S1: Figures S1–S38. Tables S1 and S2.
