# Supplementary material for: Polyester Adhesives via One-Pot, One-Step Copolymerization of Cyclic Anhydride, Epoxide, and Lactide
Source: Polymers (Basel). 2024 Sep 30;16(19):2767. doi: 10.3390/polym16192767 (PMC11479215; doi:10.3390/polym16192767)
Supplement: Supplementary file 1 [file polymers-16-02767-s001.zip › polymers-3197447-supplementary.pdf]

Supporting Information

Polyester Adhesives via One-pot, One-step Copolymerization of Cyclic Anhydride, Epoxide, and Lactide

Ryota Suzuki,[1] Toshiki Miwa,[1] Ryosuke Nunokawa,[1] Ayaka Sumi,[2] Masaru Ando,[3] Katsuaki Takahashi,[3] Akira Takagi,[3] Takuya Yamamoto,[2] Kenji Tajima,[2] Feng Li,[2] Takuya Isono,[*,2] Toshifumi Satoh[*,2,4,5]

[1] Graduate School of Chemical Sciences and Engineering, Hokkaido University, Sapporo 060-8628, Japan

[2] Division of Applied Chemistry, Faculty of Engineering, Hokkaido University, Sapporo 060-8628, Japan

[3] Toagosei Co. Ltd., Aichi 455-0026, Japan

[4] List Sustainable Digital Transformation Catalyst Collaboration Research Platform (ICReDD List-PF), Institute for Chemical Reaction Design and Discovery, Hokkaido University, Sapporo 001-0021, Japan

[5]Department of Chemical & Materials Engineering, National Central University, Taoyuan 320-0317, Taiwan

E-mail: isono.t@eng.hokudai.ac.jp

satoh@eng.hokudai.ac.jp

1. Supporting figures and tables

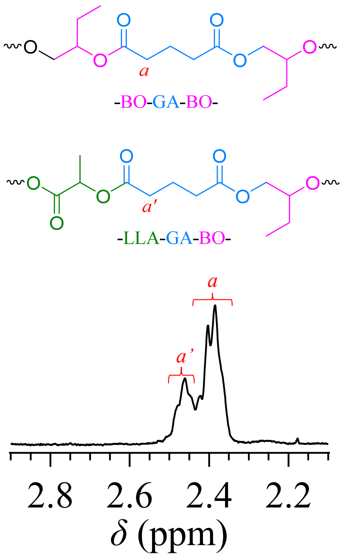

Figure S1. ^{1}H NMR spectrum of **P1** in $CDCl_3$ (400 MHz).

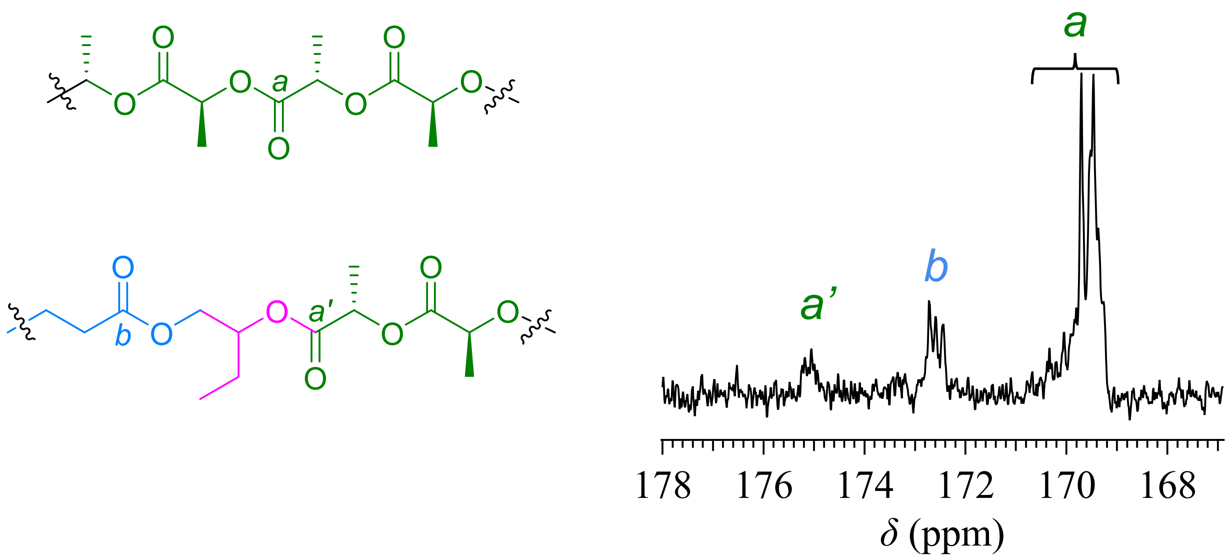

Figure S2. ^{13}C NMR spectrum of **P1** in $CDCl_3$ (100 MHz).

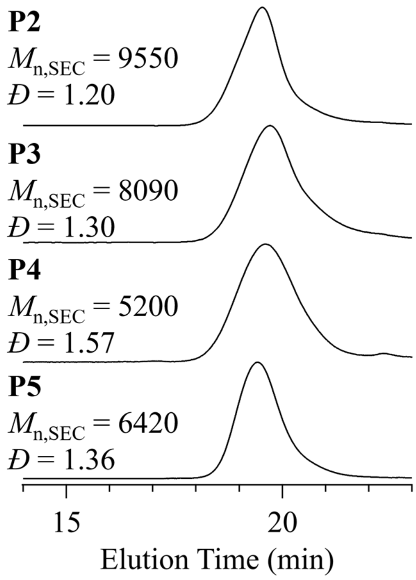

Figure S3. SEC traces of **P2–P5** (solvent, THF; flow rate, 1.0 mL min^{-1}).

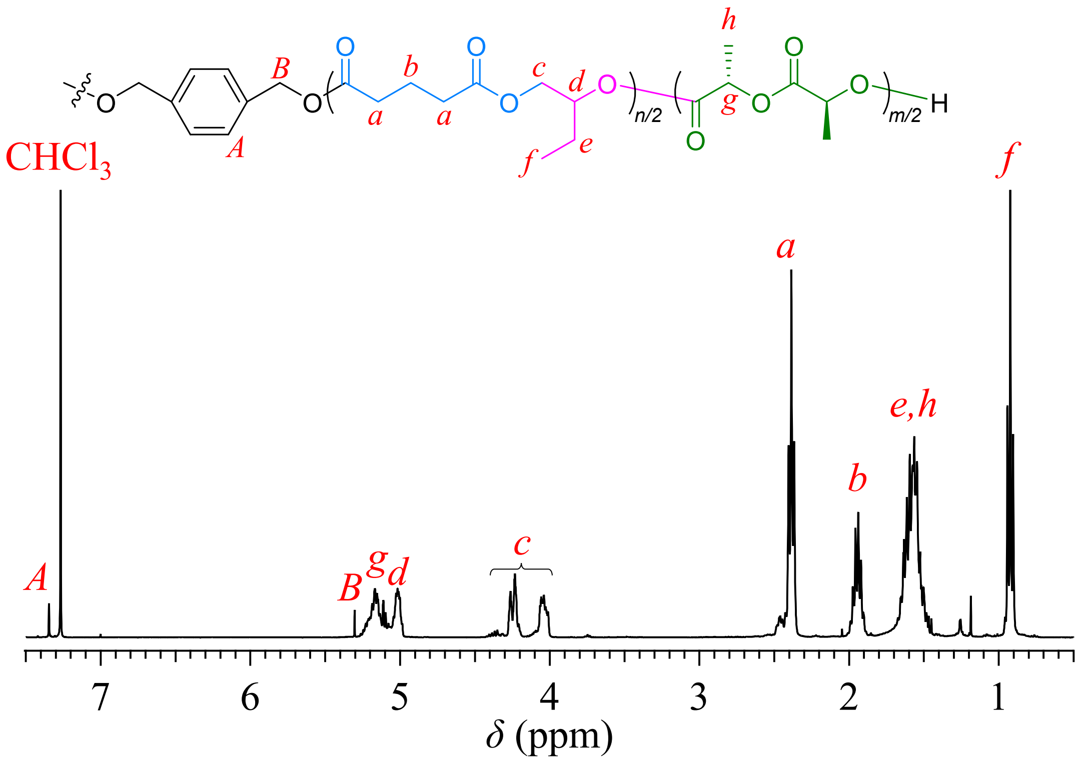

Figure S4. ^{1}H NMR spectrum of **P2** in $CDCl_3$ (400 MHz).

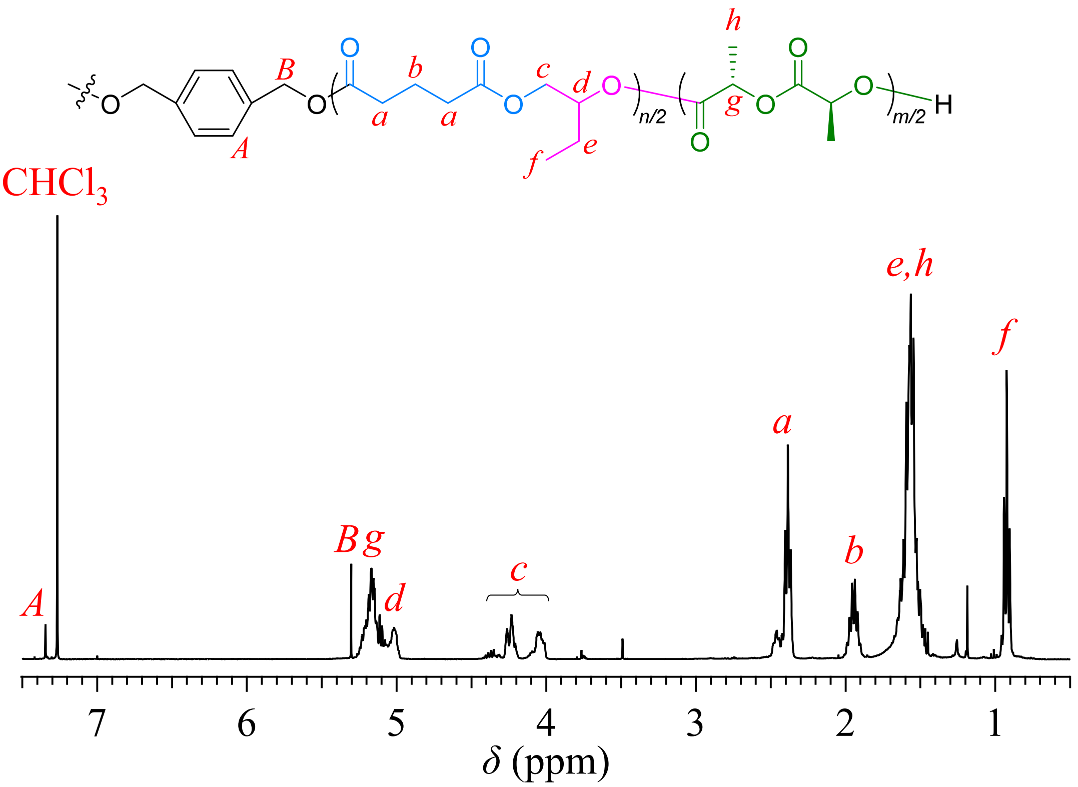

Figure S5. ^{1}H NMR spectrum of **P3** in $CDCl_3$ (400 MHz).

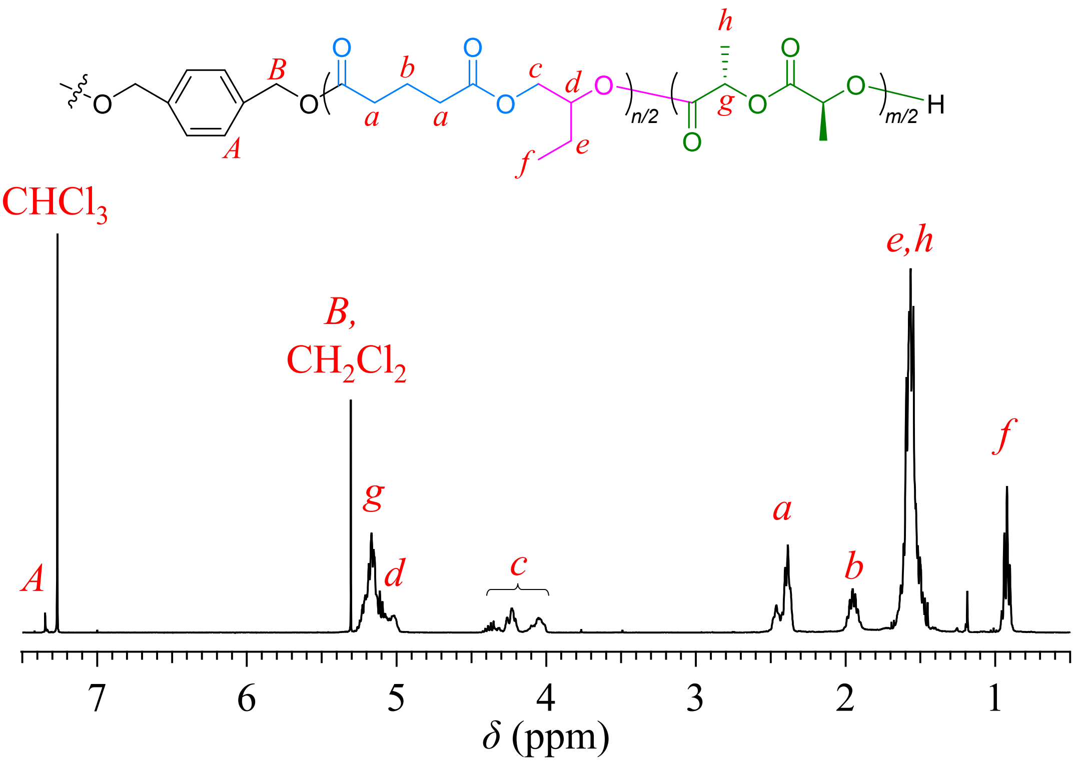

Figure S6. ^{1}H NMR spectrum of **P4** in $CDCl_3$ (400 MHz).

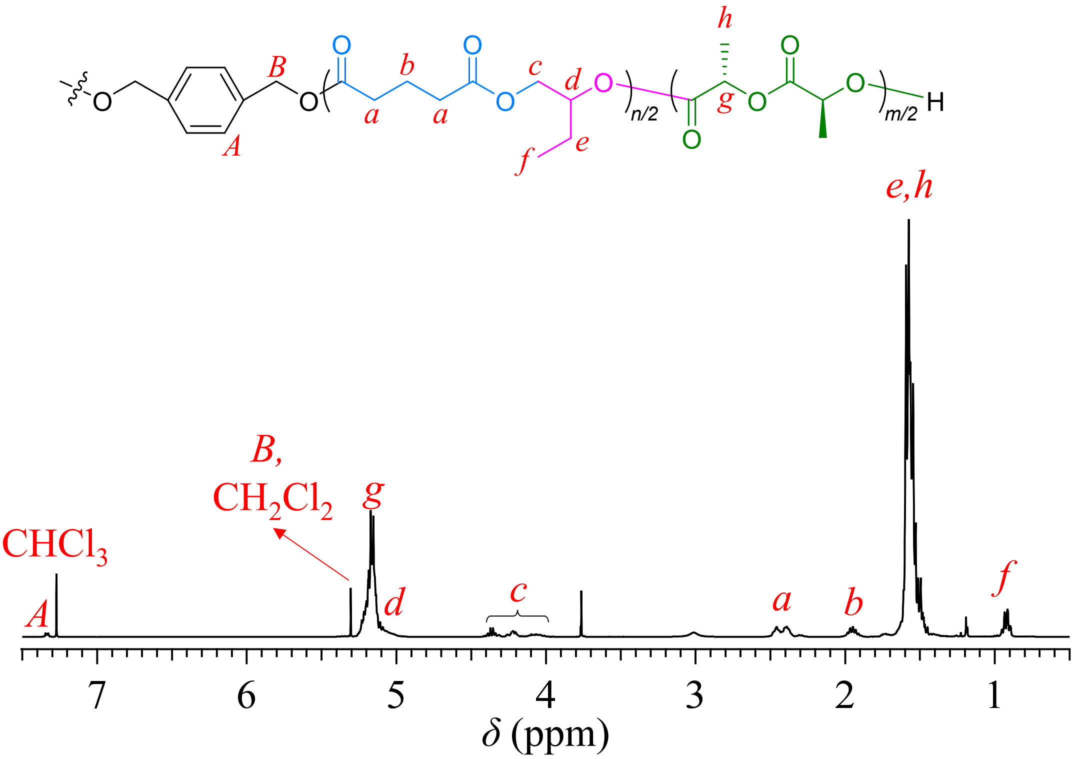

Figure S7. ^{1}H NMR spectrum of **P5** in $CDCl_3$ (400 MHz).

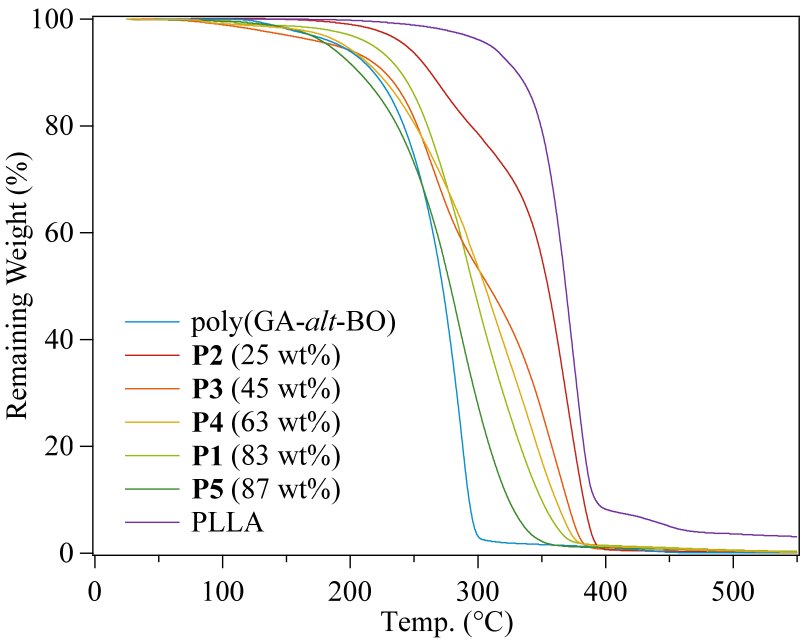

Figure S8. TGA thermograms of poly(GA-*alt*-BO) homopolymer ($M_{n,NMR}$ = 4,640), PLLA homopolymer ($M_{n,NMR}$ = 9,410), and the obtained PLLA-*tb*-poly(GA-*alt*-BO)-*tb*-PLLA (**P1**–**P5**; $M_{n,NMR}$ = 14,800–28,200, LLA ratio = 25, 45, 63, 83, and 87 wt%).

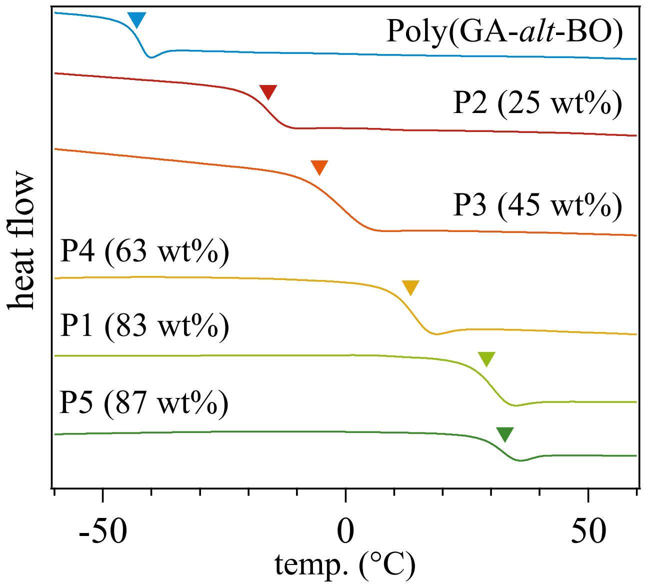

Figure S9. 1st heating DSC curves of poly(GA-*alt*-BO) homopolymer and **P1**–**P5** (heating rate, 10 °C min^{-1}; N_2 atomosphere).

Table S1. Measured and theoretical T_g for the miscible blocks

Polymer	$T_{g,measured}$/°C [a]	wt%$_{hard}$ [b]	wt%$_{soft}$ [b]	$T_{g,calculated}$/°C [c]
**P1**	29.0	83	17	35.7
**P2**	−15.6	25	75	−24.0
**P3**	−6.1	45	55	−6.2
**P4**	13.7	69	31	18.8
**P5**	32.5	87	13	40.9

[a] Measured by DSC. [b] calculated by ^{1}H NMR spectrum. [c] T_g for the sample miscribe (as calculated using the Fox equation: $1/T_{g,calculated}$ = wt%$_{hard}$/$T_{g,hard}$ + wt%$_{soft}$/$T_{g,soft}$; $T_{g,hard}$ and $T_{g,soft}$ were used 60 and −43.1 °C, respectively).

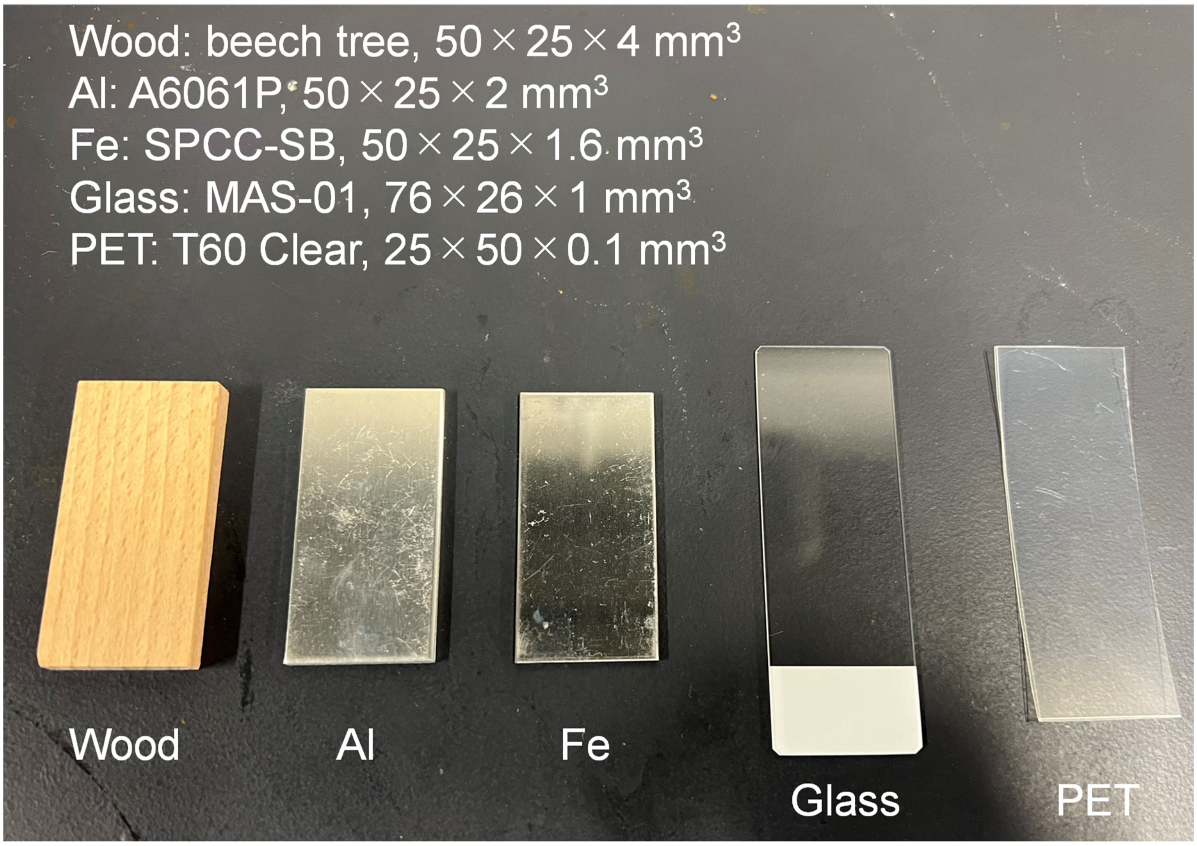

Figure S10. Digital photographs and information about adherent.

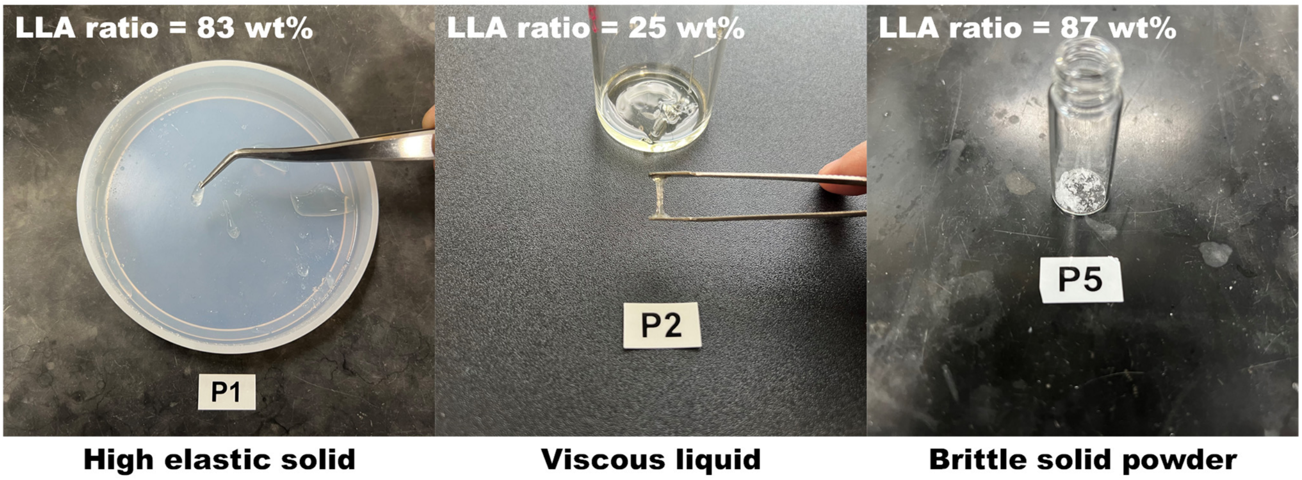

Figure S11. Digital photographs of **P1**, **P2**, and **P5**.

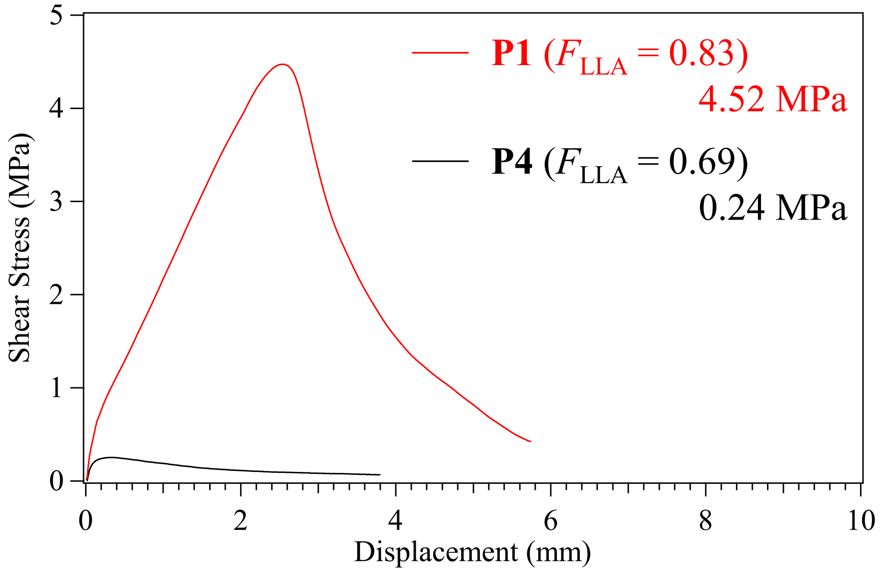

Figure S12. Lap shear tests of **P1** and **P4** using wood pieces (adherent, wood; tensile speed = 10 mm min^{-1}).

Table S2. Synthesis of PLLA and poly(GA-*alt*-BO) homopolymers [a]

sample	monomer	time (h)	conv. (%) [b]	$M_{n,theo.}$ [c]	$M_{n,NMR}$ [b]	$M_{n,SEC}$ [d]	$Đ$ [d]
PLLA [e]	LLA	1.5	80.3	6470	9410	7360	1.27
Poly(GA-*alt*-BO) [f]	GA and BO	19	>99.9	3830	4640	2560	1.61

[a] Polymerization conditions: temp., 100 °C; atmosphere, Ar; initiator, benzyl alcohol; catalyst, CsOPiv. [b] Calculated from (M.W. of initiator) + $[\text{monomer}]_0/[\text{initiator}]_0$ × (M.W. of monomer). [c] Determined by ^{1}H NMR in $CDCl_3$. [d] Determined by SEC in THF using polystyrene standards. [e] $[\text{CsOPiv}]/[\text{initiator}]_0/[\text{LLA}]_0$ = 1/1/55. [f] $[\text{CsOPiv}]/[\text{initiator}]_0/[\text{GA}]_0/[\text{BO}]_0$ = 1/1/20/80.

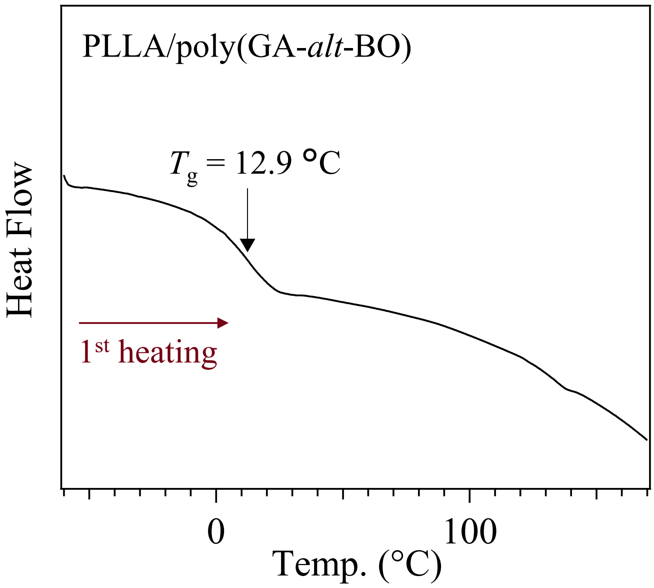

Figure S13. 1st heating DSC curves of polymer blend (PLLA/poly(GA-*alt*-BO) = 83/17 (weight ratio); 1st heating, 10 °C/min, N_2 atmosphere).

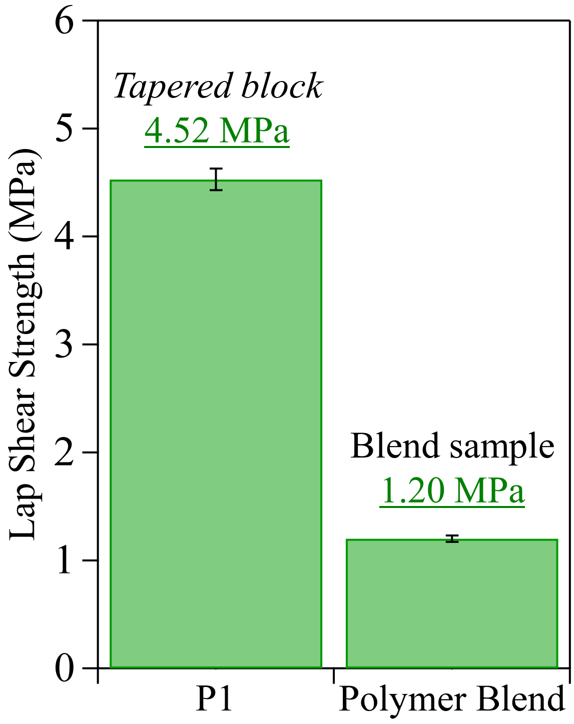

Figure S14. Lap shear strengths of **P1** and polymer blend (PLLA/poly(GA-*alt*-BO) = 83/17 (weight fraction)).

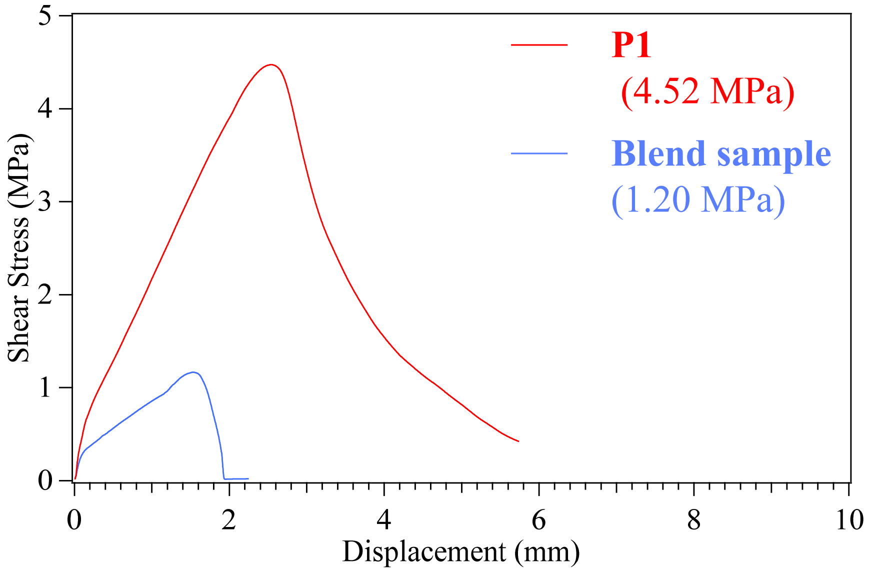

Figure S15. Lap shear tests of **P1** and polymer blend sample of PLLA and poly(GA-*alt*-BO) (PLLA/poly(GA-*alt*-BO) = 83/17 (weight ratio)) using wood pieces (adherent, wood; tensile speed = 10 mm min^{-1}).

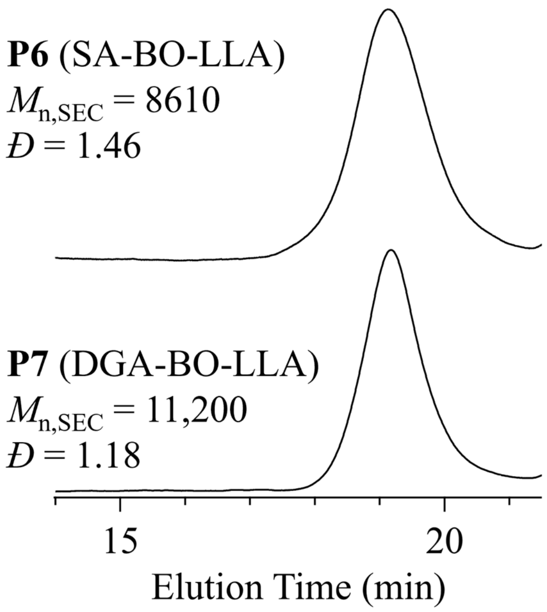

Figure S16. SEC traces of **P6–P7** (solvent, THF; flow rate, 1.0 mL min^{-1}).

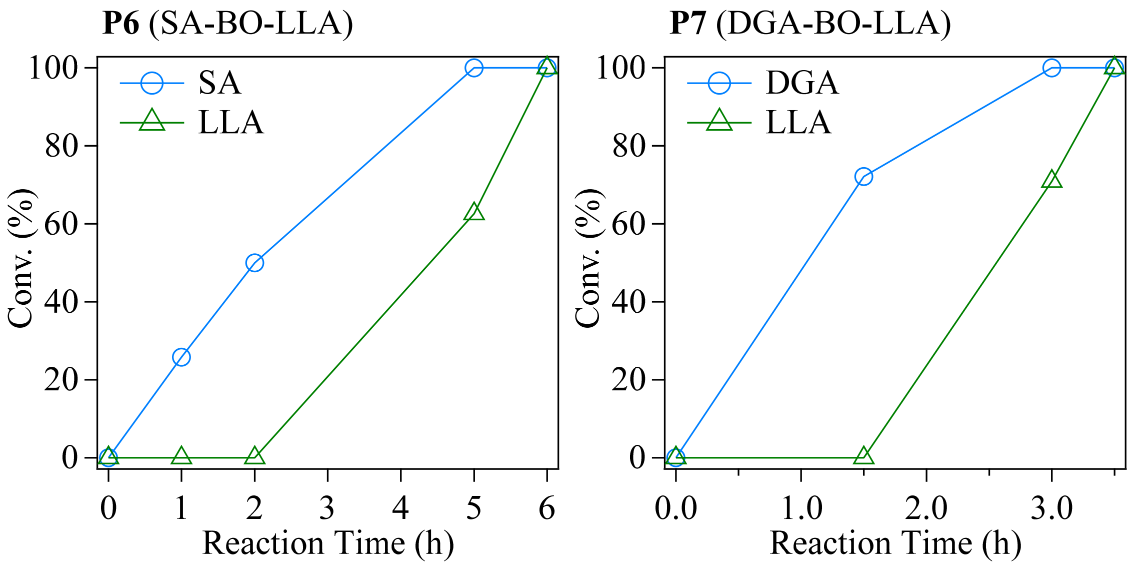

Figure S17. Time to conv. plots for the synthesis of **P6–P7** monitored by ^{1}H NMR.

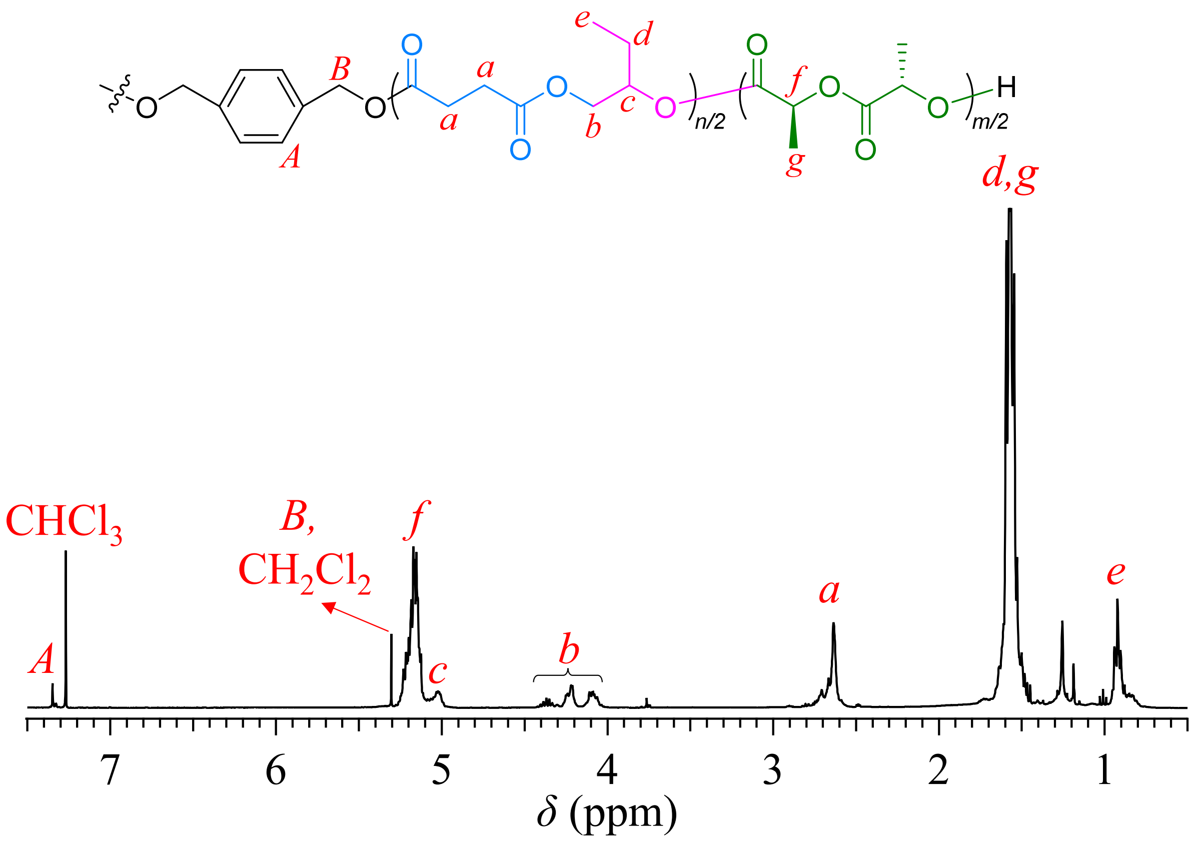

Figure S18. ^{1}H NMR spectrum of **P6** in CDCl3 (400 MHz).

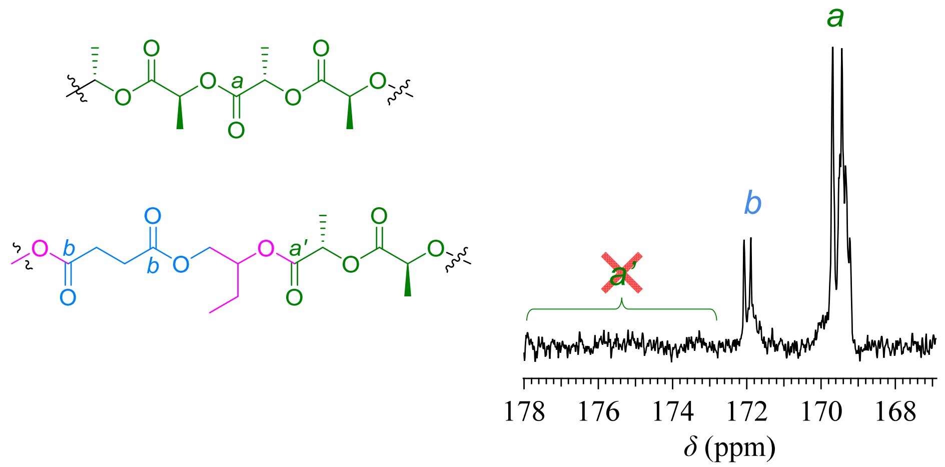

Figure S19. ^{13}C NMR spectrum of **P6** in CDCl3 (100 MHz).

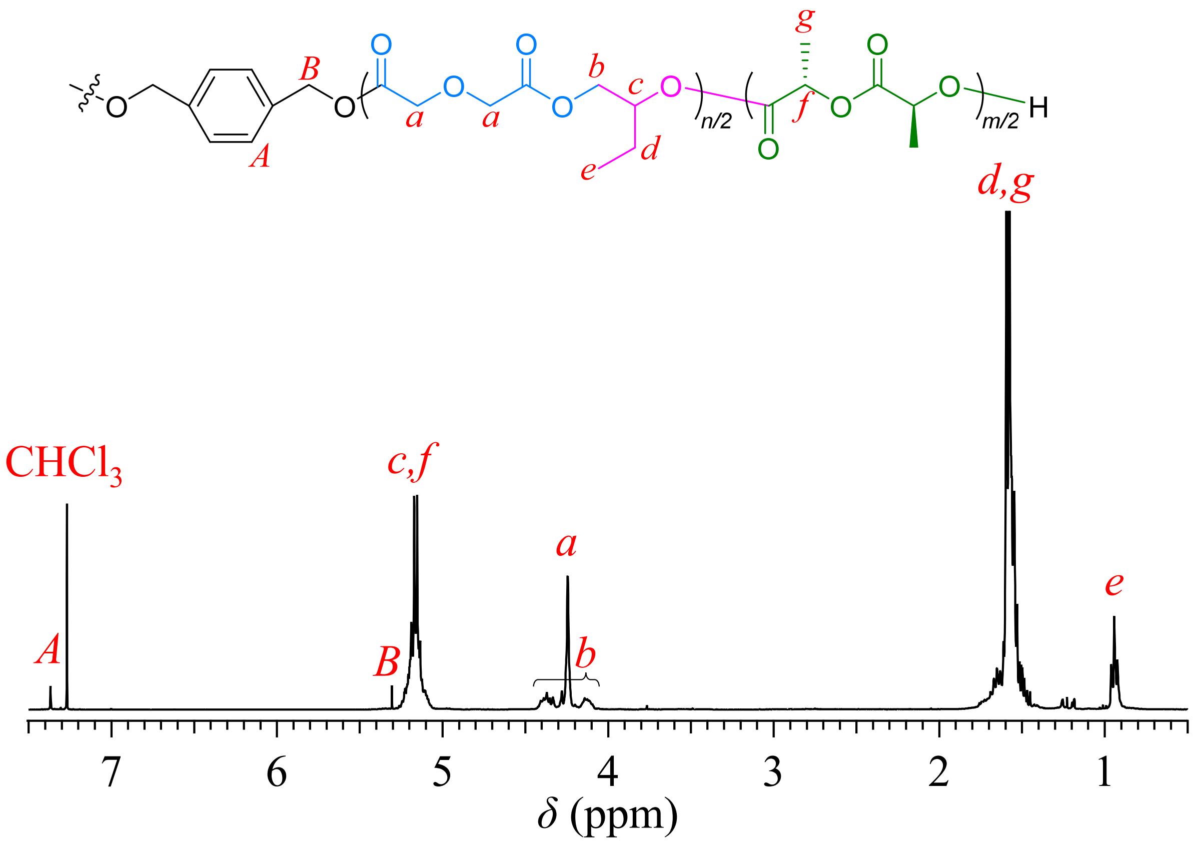

Figure S20. ^{1}H NMR spectrum of **P7** in $CDCl_3$ (400 MHz).

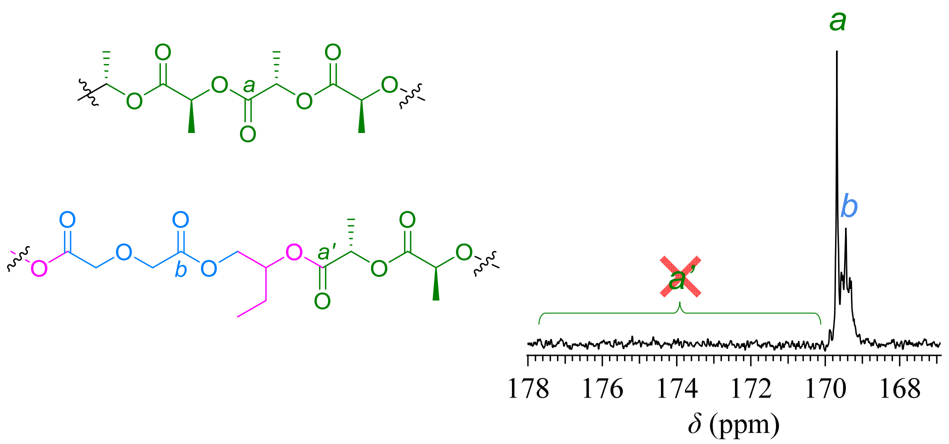

Figure S21. ^{13}C NMR spectrum of **P7** in $CDCl_3$ (100 MHz).

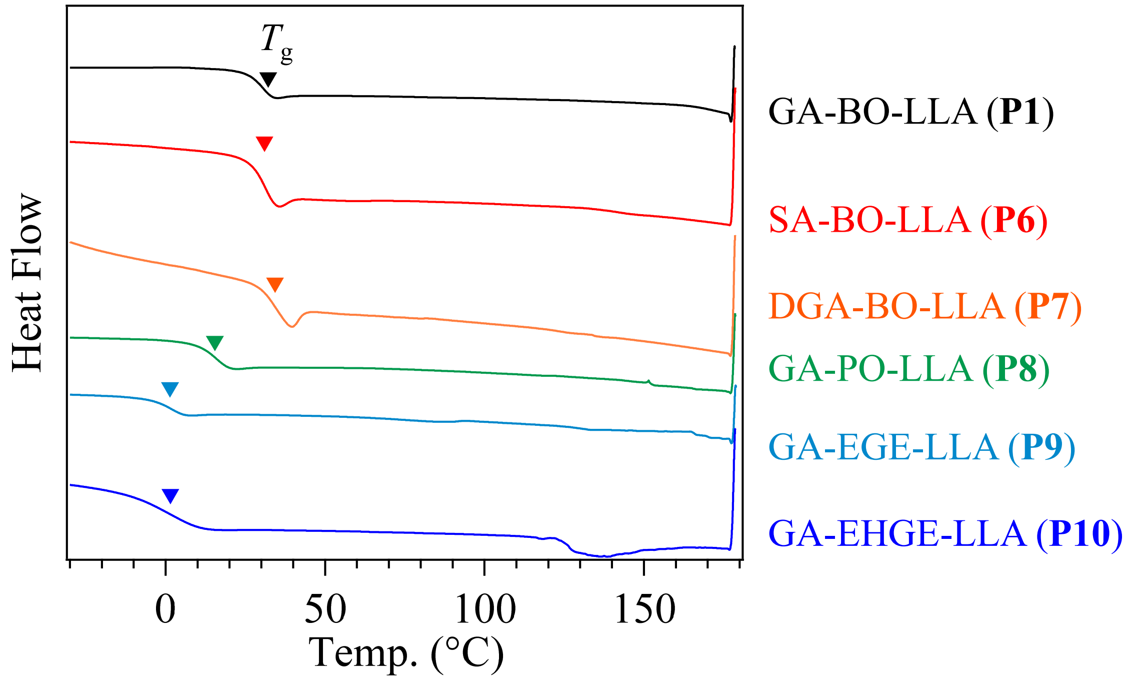

Figure S22. 1st heating DSC curves of **P1**, **P6–P10** (1st heating, 10 °C min^{-1}, N_2 atmosphere).

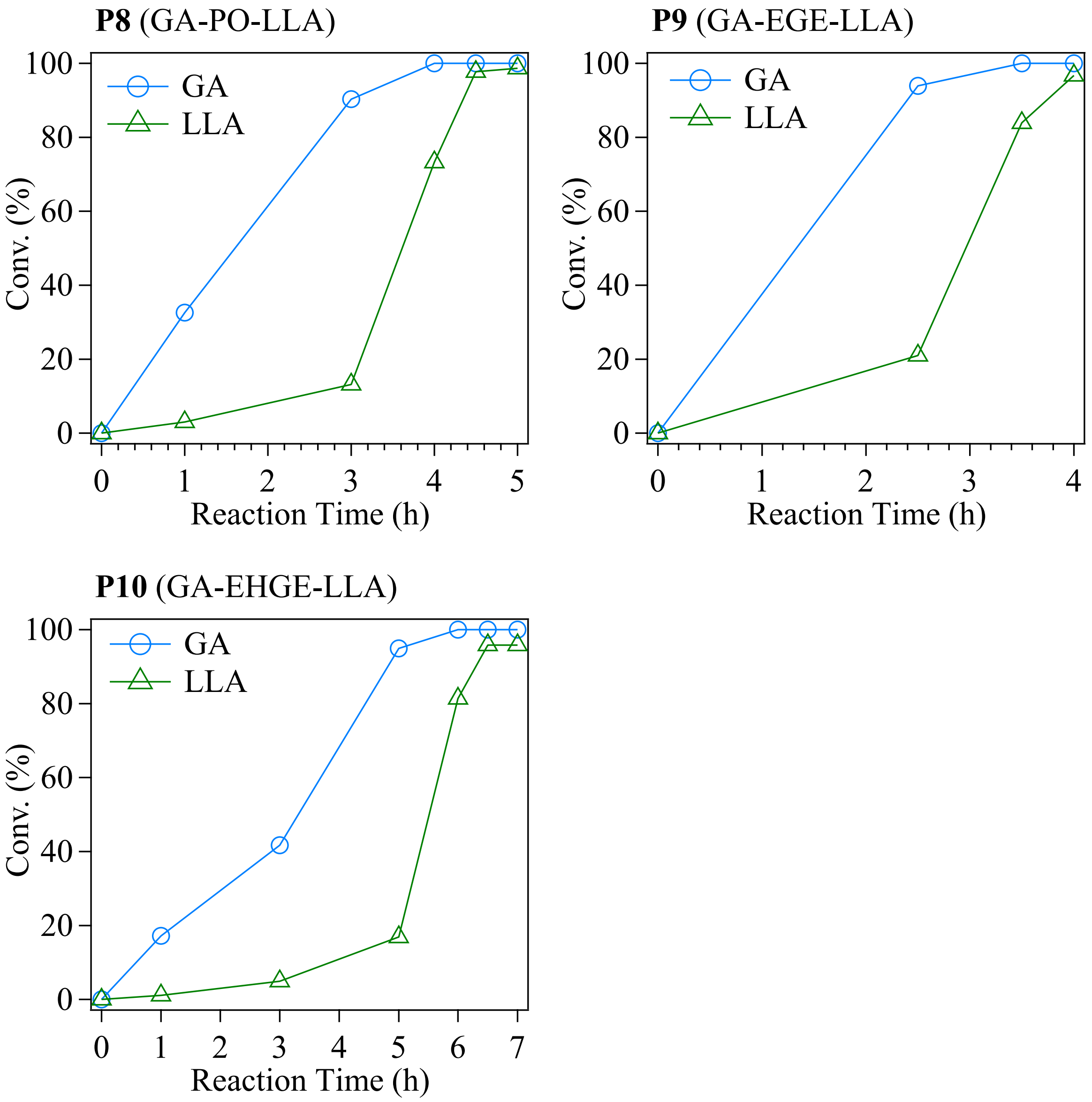

Figure S23. Time to conv. plots for the synthesis of **P8–P10** monitored by ^{1}H NMR.

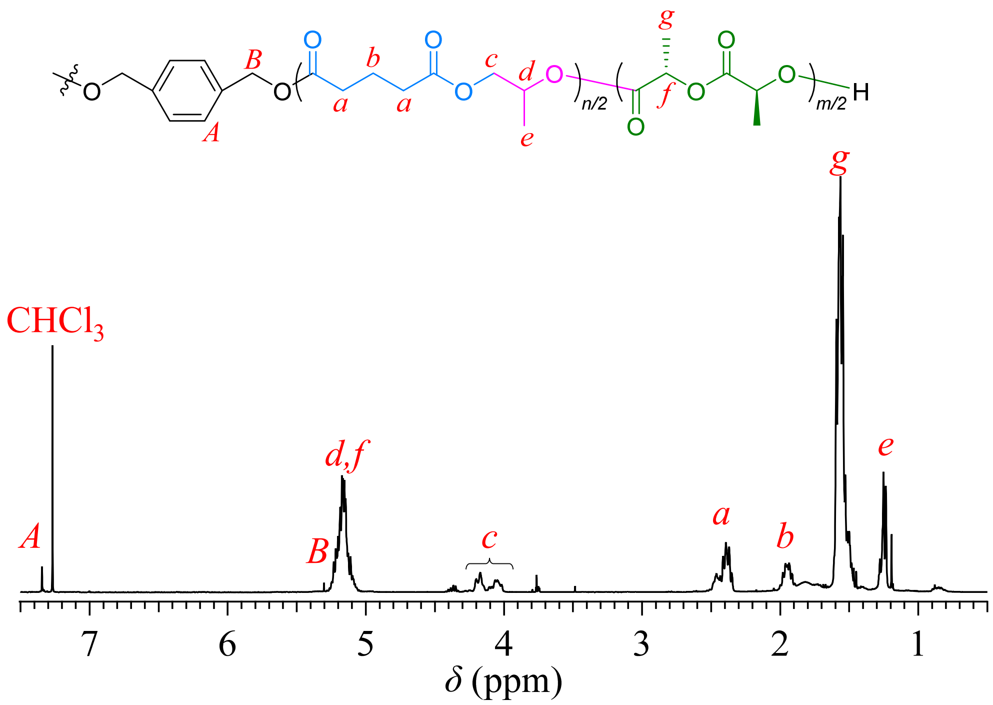

Figure S24. ^{1}H NMR spectrum of PLLA-*tb*-poly(GA-*alt*-PO)-*tb*-PLLA in $CDCl_3$ (**P8** in **Table 1**; 400 MHz).

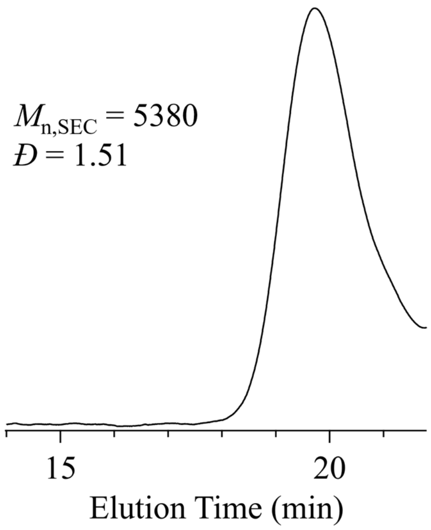

Figure S25. SEC trace of PLLA-*b*-poly(GA-*alt*-PO)-*b*-PLLA (**P8** in **Table 1**; eluent, THF; flow rate, 1.0 mL min^{-1}).

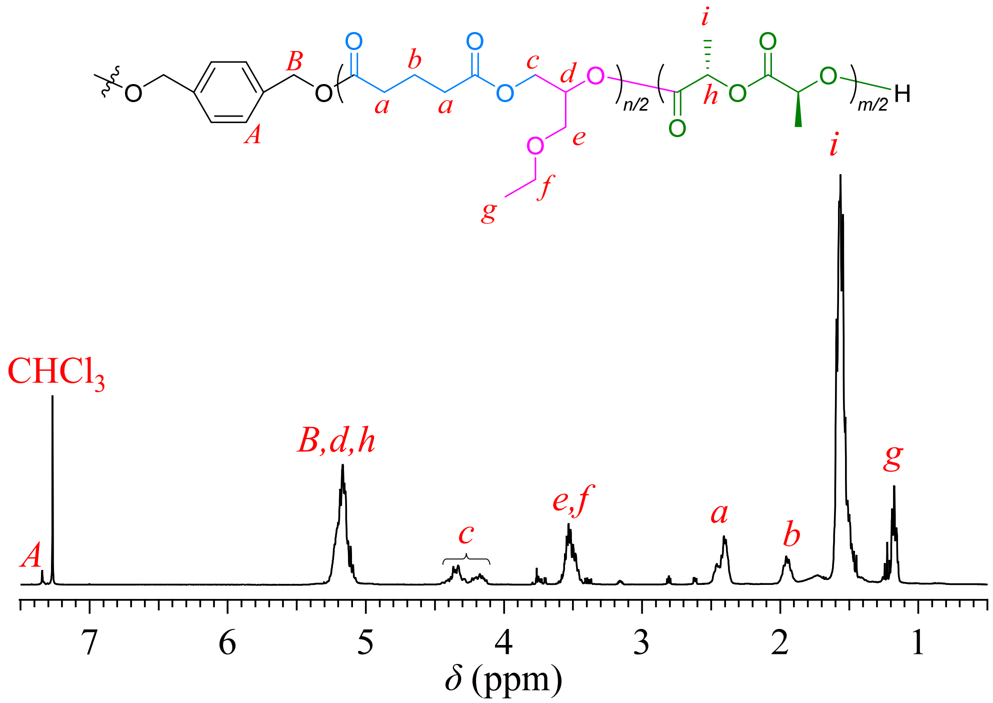

Figure S26. ^{1}H NMR spectrum of PLLA-*tb*-poly(GA-*alt*-EGE)-*tb*-PLLA in $CDCl_3$ (**P9** in **Table 1**; 400 MHz).

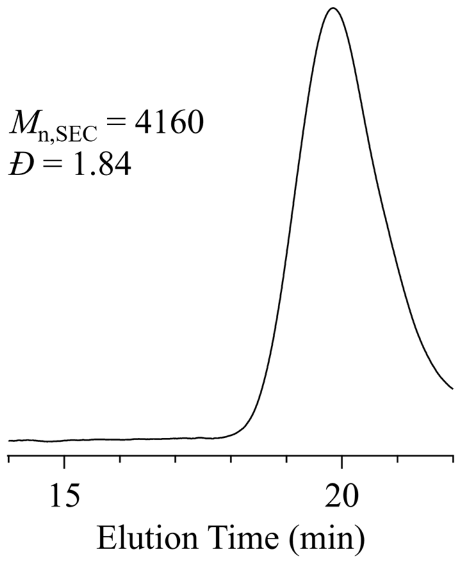

Figure S27. SEC trace of PLLA-*b*-poly(GA-*alt*-EGE)-*b*-PLLA (**P9** in **Table 1**; THF; flow rate, 1.0 mL min^{-1}).

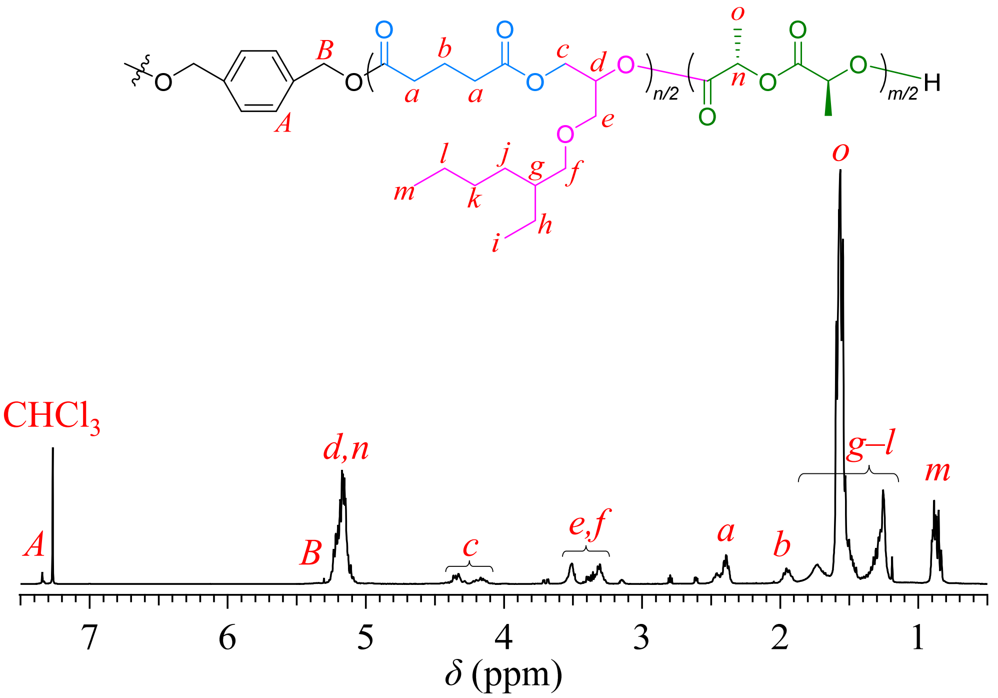

Figure S28. ^{1}H NMR spectrum of PLLA-*b*-poly(GA-*alt*-EHGE)-*b*-PLLA in $CDCl_3$ (**P10** in **Table 1**; 400 MHz).

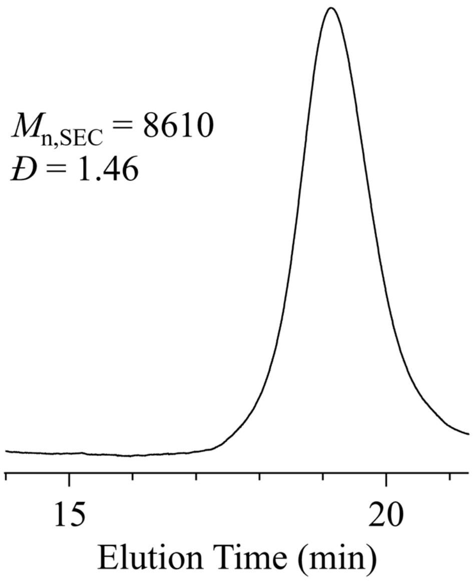

Figure S29. SEC trace of PLLA-*b*-poly(GA-*alt*-EHGE)-*b*-PLLA (**P10** in **Table 1**; eluent, THF; flow rate, 1.0 mL min^{-1}).

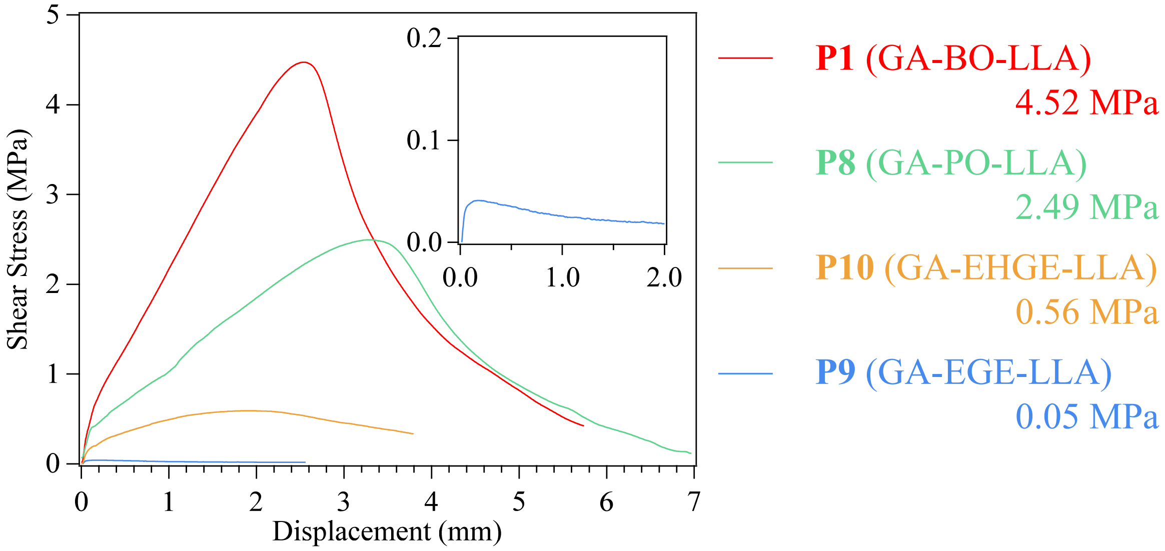

Figure S30. Lap shear tests of PLLA-*tb*-poly(anhydride-*alt*-epoxide)-*tb*-PLLA using wood test pieces (**P1**, **P8**–**P10** in **Table 1**; adherent, wood; tensile speed = 10 mm min^{-1}).

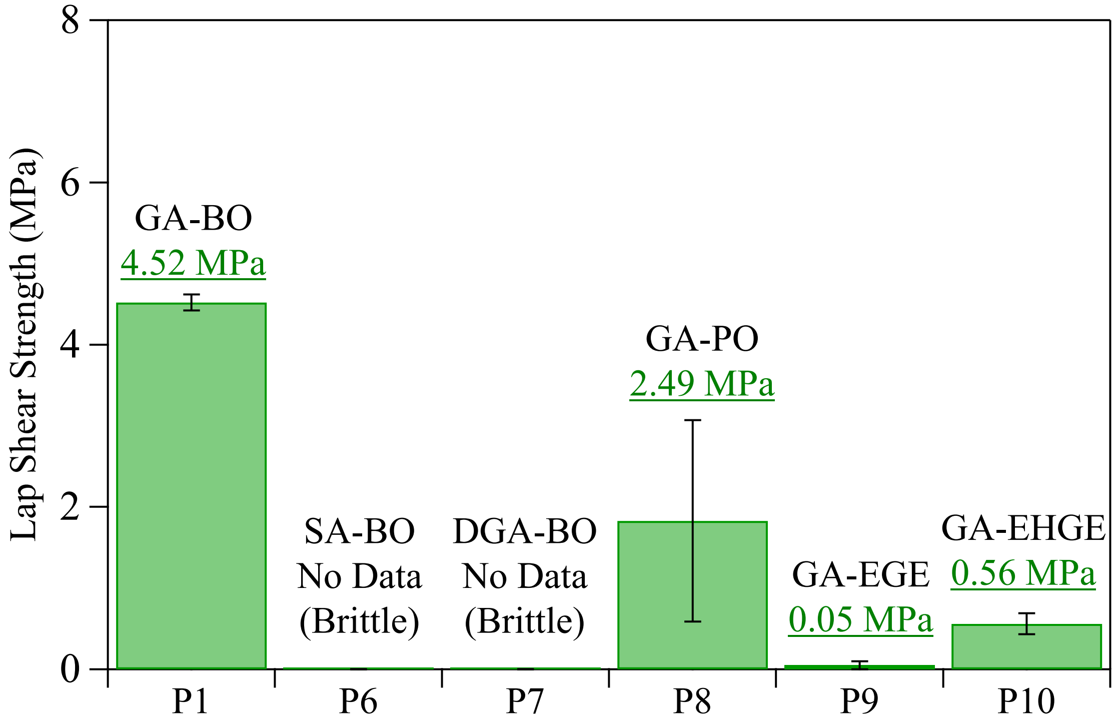

Figure S31. Lap shear strengths of BCPs with various repeating unit structures (adherent, wood; tensile speed = 10 mm min^{-1}; n = 3).

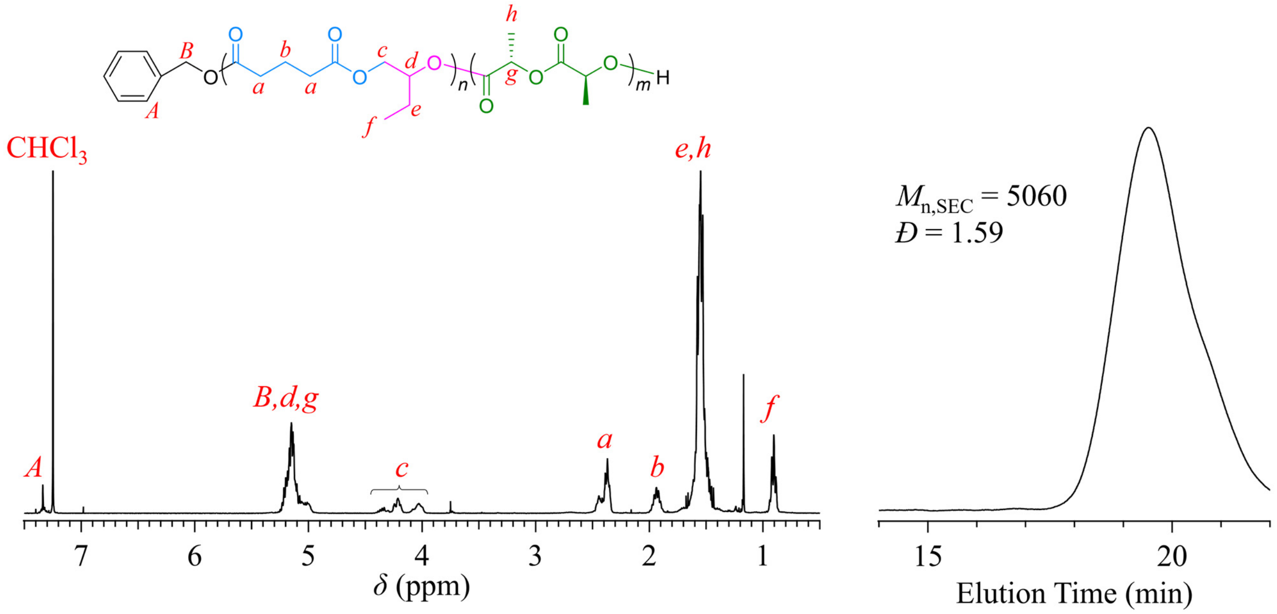

Figure S32. ^{1}H NMR spectrum ($CDCl_3$, 400 MHz; left panel) and SEC trace (eluent, THF; flow rate, 1.0 mL min^{-1}; right panel) of poly(GA-*alt*-BO)-*tb*-PLLA (**P11** in **Table 2**).

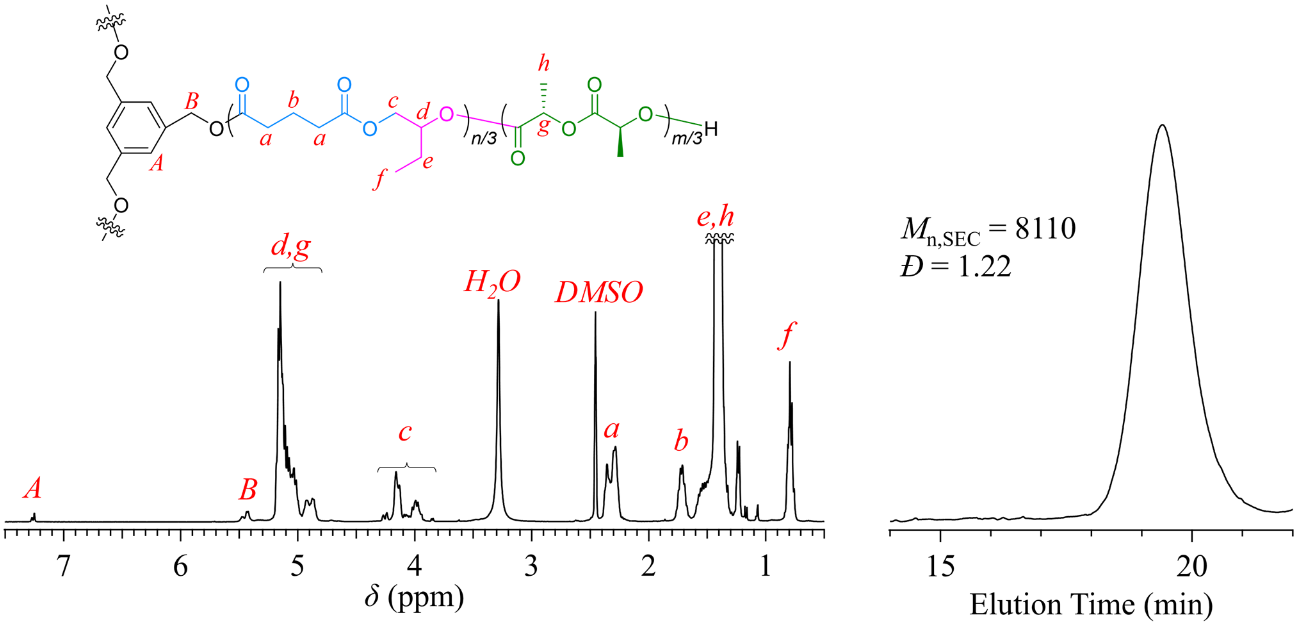

Figure S33. ^{1}H NMR spectrum (DMSO-*d*6, 400 MHz; left panel) and SEC trace (eluent, THF; flow rate, 1.0 mL min^{-1}; right panel) of (poly(GA-*alt*-BO)-*tb*-PLLA)3 (**P12** in **Table 2**).

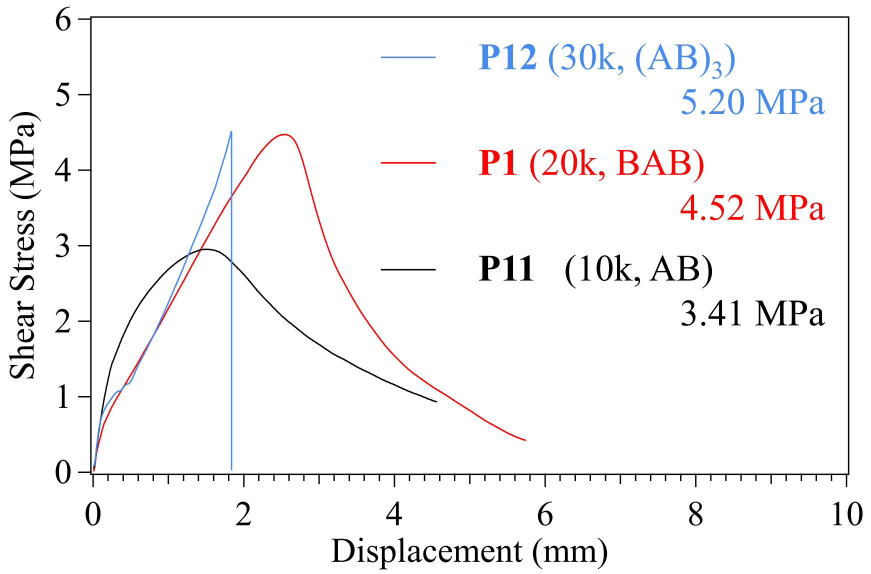

Figure S34. Lap shear tests of **P1**, **P11**, and **P12** using wood pieces (adherent, wood; tensile speed = 10 mm min^{-1}).

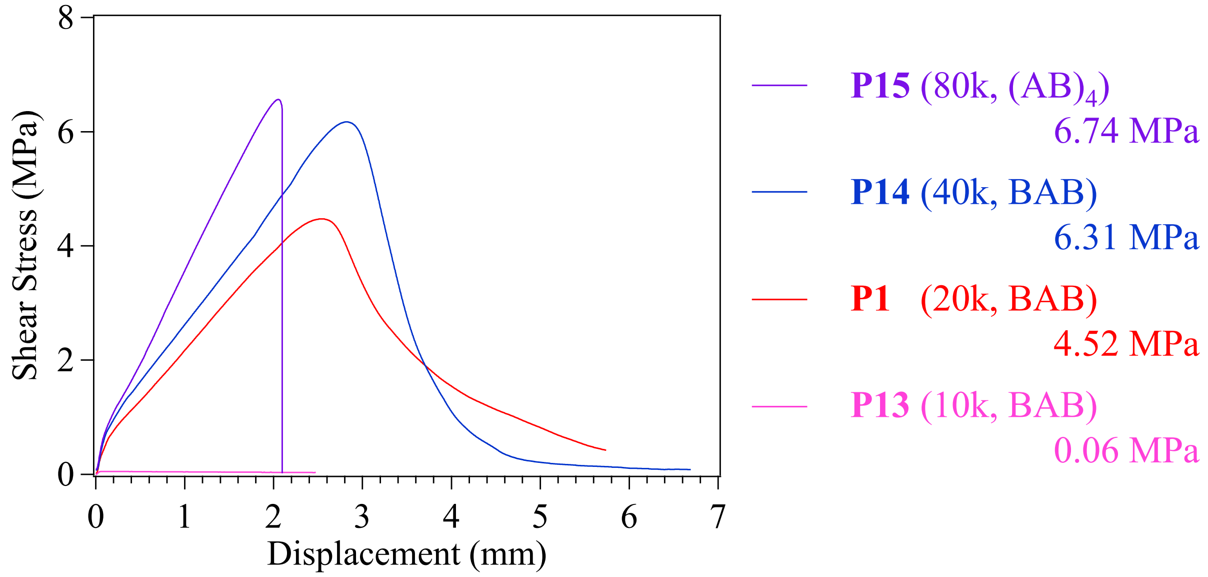

Figure S35. Lap shear tests of **P1** and **P13–P15** using wood pieces (adherent, wood; tensile speed = 10 mm min^{-1}).

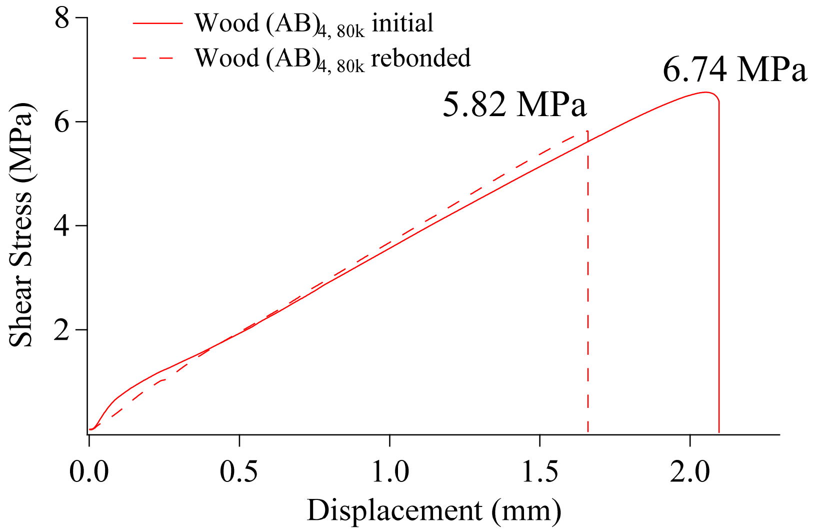

Figure S36. Lap shear strength of **P15** on Wood adherent during recycling.

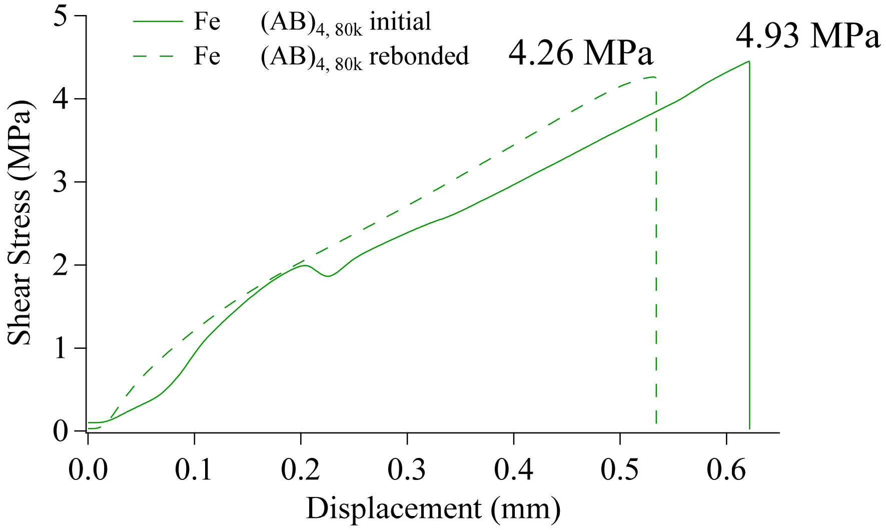

Figure S37. Lap shear strength of **P15** on Fe adherent during recycling.

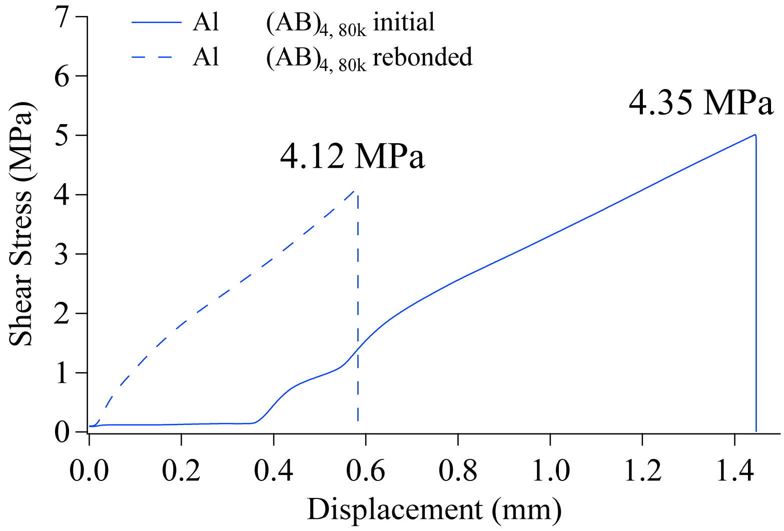

Figure S38. Lap shear strength of **P15** on Al adherent during recycling.
